# A Broadly Conserved G-Protein-Coupled Receptor Kinase Phosphorylation Mechanism Controls *Drosophila* Smoothened Activity

**DOI:** 10.1371/journal.pgen.1004399

**Published:** 2014-07-10

**Authors:** Dominic Maier, Shuofei Cheng, Denis Faubert, David R. Hipfner

**Affiliations:** 1 Institut de recherches cliniques de Montréal (IRCM), Montreal, Quebec, Canada; 2 Department of Anatomy & Cell Biology, McGill University, Montreal, Quebec, Canada; 3 Proteomics Core Facility, IRCM, Montreal, Quebec, Canada; 4 Department of Medicine, Université de Montréal, Montreal, Quebec, Canada; Harvard Medical School, Howard Hughes Medical Institute, United States of America

## Abstract

Hedgehog (Hh) signaling is essential for normal growth, patterning, and homeostasis of many tissues in diverse organisms, and is misregulated in a variety of diseases including cancer. Cytoplasmic Hedgehog signaling is activated by multisite phosphorylation of the seven-pass transmembrane protein Smoothened (Smo) in its cytoplasmic C-terminus. Aside from a short membrane-proximal stretch, the sequence of the C-terminus is highly divergent in different phyla, and the evidence suggests that the precise mechanism of Smo activation and transduction of the signal to downstream effectors also differs. To clarify the conserved role of G-protein-coupled receptor kinases (GRKs) in Smo regulation, we mapped four clusters of phosphorylation sites in the membrane-proximal C-terminus of *Drosophila* Smo that are phosphorylated by Gprk2, one of the two fly GRKs. Phosphorylation at these sites enhances Smo dimerization and increases but is not essential for Smo activity. Three of these clusters overlap with regulatory phosphorylation sites in mouse Smo and are highly conserved throughout the bilaterian lineages, suggesting that they serve a common function. Consistent with this, we find that a C-terminally truncated form of *Drosophila* Smo consisting of just the highly conserved core, including Gprk2 regulatory sites, can recruit the downstream effector Costal-2 and activate target gene expression, in a Gprk2-dependent manner. These results indicate that GRK phosphorylation in the membrane proximal C-terminus is an evolutionarily ancient mechanism of Smo regulation, and point to a higher degree of similarity in the regulation and signaling mechanisms of bilaterian Smo proteins than has previously been recognized.

## Author Summary

Hedgehog proteins are critical regulators of embryonic tissue growth and organization in species ranging from flies to humans. Binding of the secreted Hh protein to its receptor at the surface of cells triggers an intracellular signal that is initiated by Smoothened (Smo). Upon exposure of cells to Hh, Smo becomes active and signals through a series of downstream proteins to regulate gene expression. Although Smo proteins in flies and mammals are similar, the critical regions involved in activation and signal initiation differ between the two, implying that different mechanisms have evolved in different organisms. Using the fruit fly as a model organism, we identified regions in Smo that are phosphorylated by a protein kinase called Gprk2 to enhance Smo activity. These phosphorylation sites overlap with previously identified sites in mouse Smo and are conserved in Smo proteins in many animals. Phosphorylation at these sites regulates the recruitment of Costal2 to Smo, a critical step in signal initiation, through a region of the protein that is also highly conserved. Our results indicate that Gprk2 phosphorylation represents an evolutionarily ancient and conserved mechanism for regulating Smo activity, and suggest that Smo regulation and signaling are more similar between different species than previously thought.

## Introduction

The Smoothened (Smo) family of seven-pass transmembrane proteins initiate cytoplasmic Hedgehog (Hh) signaling. Smo proteins are activated by multisite phosphorylation in the cytoplasmic C-terminal tail, which counteracts the electrostatic effects of adjacent clusters of positively charged residues thought to maintain the protein in an inactive conformation. Upon phosphorylation, Smo undergoes a conformational change, dimerizes, and accumulates at the plasma membrane (*Drosophila*) or primary cilium (mammals), and activates downstream signaling, leading to stabilization of Ci/Gli family transcription factors and Hh target gene expression [1]–[3]. Smo is phosphorylated in a graded manner, with higher levels of Hh ligand inducing more extensive phosphorylation and thereby driving expression of higher-threshold target genes [2], [4].

Although the extracellular N-terminus and seven-transmembrane regions of Smo are highly conserved, only the first 100 amino acids of the cytoplasmic C-terminus is broadly conserved. The more distal C-terminus, in many cases hundreds of amino acids long, is completely divergent in different phyla. Because they target different portions of the C-terminus, the phosphorylation mechanisms regulating Smo differ fundamentally between invertebrates and vertebrates. In *Drosophila*, phosphorylation at three clusters of Protein kinase A (PKA) and Casein kinase I (CKI) sites located in the Smo autoinhibitory domain (SAID) is necessary and sufficient for activation [5]–[7]. However, neither the SAID nor the critical PKA sites in *Drosophila* Smo are conserved in vertebrate Smo proteins; instead G-protein-coupled receptor kinase (GRK) 2 and CKI are the principal kinases that activate vertebrate Smo proteins by phosphorylating them at a different set of sites [2].

The means by which Smo engages the downstream signaling machinery through its C-terminus also appears to have diverged. In *Drosophila* and vertebrates, Smo binds to the downstream effector Cos2 and its orthologue Kif7, respectively [8]–[10]. In addition to a negative role in Hh signaling, Cos2 and Kif7 are required for full activation of the pathway [11]–[13], likely in the case of Cos2 because it helps to recruit the kinase Fused to Smo and to activate it [14]. Previous studies localized two separate binding sites for Cos2 in the *Drosophila* Smo C-terminus [8], [9], neither of which is conserved in vertebrates. Given that *Drosophila* and vertebrate Smo signal through similar effectors [3], this divergence has been puzzling [15].

GRKs are also implicated in Smo activation in *Drosophila*. Gprk2, one of the two *Drosophila* GRKs, is required for Smo to drive high-threshold Hh target gene expression [16]–[18]. Two pairs of Gprk2 phosphorylation sites (called GPS1 and GPS2) have been mapped to the non-conserved portion of the Smo C-terminus, with phosphorylation at GPS1 suggested to contribute to the charge mechanism that overcomes inhibition by the SAID [18]. Dimerization of the kinase itself was also suggested to help promote Smo dimerization and activation in a catalytic-activity-independent manner [18]. On the other hand, loss of *gprk2* causes a reduction in cyclic AMP (cAMP) levels, affecting PKA-dependent Smo activation. Target gene expression can be largely rescued in *gprk2* mutants by increasing cAMP levels, suggesting that this indirect effect of Gprk2 on Smo plays an important role in Hh pathway function [19].

To further explore the effects of direct Gprk2 phosphorylation on Smo activity, we mapped four new clusters of Gprk2-dependent phosphorylation sites in the cytoplasmic C-terminus. We find that mutation of these sites to non-phosphorylatable residues reduces Smo dimerization and its ability to promote Hh target gene expression. Phosphomimetic mutations bypass the requirement for Gprk2. Importantly, the phosphosites we mapped overlap with CKI/GRK sites mapped in the mouse Smo C-terminus, and are remarkably well-conserved in Smo orthologues throughout the bilaterian lineages. We demonstrate that the evolutionarily conserved core of Smo is a functional, GRK-regulated protein that is sufficient to activate downstream signaling, suggesting that all bilaterian Smo proteins share a common regulatory and signaling mechanism.

## Results

We previously reported that Gprk2 affects phosphorylation of Smo at sites other than GPS1 and GPS2 [19]. Even when using a PKA- and CKI-phosphomimetic form of Smo, Smo^SD^ [5] to circumvent the effects of *gprk2* depletion on cAMP levels, there was a substantial reduction of Smo phosphorylation (detected by increased mobility in SDS-PAGE) upon dsRNA-mediated depletion of Gprk2 that could not be accounted for by the GPS sites (Figure S1A). Previous analysis suggested that mutation of the GPS sites to non-phosphorylatable Ala residues (in Smo^SD.GPSA12^) had only a small effect on Smo signaling activity [18]. We found that Smo^SD^ and Smo^SD.GPSA12^ had similar ability to drive expression of the highest-threshold Hh target gene, anterior *engrailed* (*en*), *in vivo* (Figure S1B and C). These results suggest that the GPS sites account for at most a small fraction of functionally important Gprk2 phosphorylation sites in Smo.

**Figure 1:**
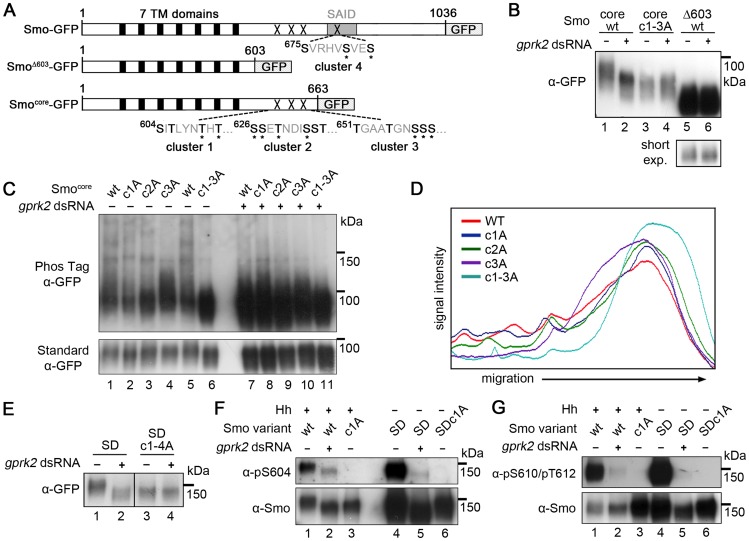
Mapping of Gprk2 phosphorylation sites. (*A*) Schematics of full-length and truncated Smo proteins (not to scale). *Black boxes*: transmembrane domains. *Grey box*: SAID domain. *X's*: Gprk2 phosphorylation clusters. Sequences within each cluster are shown with potential phosphorylation sites bolded. *Asterisks*: residues previously identified as phosphosites [6]. (*B*) Immunoblot analysis of truncated GFP-tagged Smo molecules, expressed in cells treated +/− *gprk2* dsRNA. Ala substitution of all three Ser/Thr clusters eliminates Gprk2-dependent phosphorylation of Smo. (*C*) Immunoblot analysis of Smo^core^-GFP or mutants with one or all three Gprk2 phosphorylation sites mutated to Ala, expressed in cells +/− *gprk2* dsRNA. Samples were separated on Phos-Tag-conjugated (*top panel*) or standard (*bottom panel*) SDS-PAGE gels. (*D*) Plot of signal intensity versus migration distance for the immunoblot of Smo^core^-GFP mutants (−*gprk2* dsRNA conditions, left side of panel *C*). Ala substitutions within each Ser/Thr cluster increased Smo mobility, suggesting sites in all three clusters are phosphorylated. (*E*) Immunoblot analysis of Smo^SD^ or Smo^SD.c1-4A^ expressed in cells treated +/− *gprk2* dsRNA. Ala substitution of all four Ser/Thr clusters eliminates Gprk2-dependent phosphorylation of full-length Smo. (*F* and *G*) Immunoblot of Smo^WT^, Smo^c1A^, Smo^SD^, or Smo^SD.c1a^, expressed in cells +/− *gprk2* dsRNA. Blots were probed with Smo phosphospecific antisera: anti-pS^604^ (*F*) or anti-pT^610^/pT^612^ (*G*). Phosphorylation at both sites was strongly decreased by *gprk2* depletion.

In order to localize the functionally important sites, we tested the ability of Gprk2 to phosphorylate a series of C-terminally truncated, GFP-tagged forms of Smo in cells (Figure 1A). Smo truncated at amino acid 663 (Smo^core^-GFP), which retains the highly conserved core of the protein but lacks more than 75% of the cytoplasmic tail including all three clustered PKA/CKI sites, still shifted in response to *gprk2* depletion (Figure 1B, lanes 1 and 2). Further truncation to amino acid 603 (Smo^Δ603^-GFP) eliminated this response (Figure 1B, lanes 5 and 6). The intervening 60 amino acids include 15 Ser/Thr residues, nine of which were previously identified as phosphosites by mass spectrometry [6], grouped into three clusters (Figure 1A). Mutation of all three Ser/Thr clusters in Smo^core^ to non-phosphorylatable Ala residues (Smo^core.c1-3A^-GFP) eliminated Gprk2-dependent phosphorylation (Figure 1B, lanes 3 and 4). When fractionated in SDS-PAGE gels containing Phos-Tag, which specifically retards the migration of phosphorylated proteins [20], Smo^core^-GFP migrated as a high molecular weight smear extending from ∼100 kDa to the top of the gel (Figure 1C, lanes 1 and 5; quantified in Figure 1D). After Gprk2 depletion, Smo^core^-GFP ran as a more discrete, faster-migrating band (Figure 1C, lane 7), confirming that retardation of Smo^core^-GFP migration is largely due to Gprk2-dependent phosphorylation. Mutation of any one of the three phosphorylation clusters had an intermediate effect on Smo mobility, increasing the proportion of protein in the faster-migrating fraction (Figure 1C, lanes 2–4 and Figure 1D), whereas Smo^core.c1-3A^-GFP co-migrated with Smo^core^-GFP from Gprk2-depleted cells (Figure 1C, lanes 6 and 7). These results suggest that Gprk2 phosphorylates residues in each of the three clusters. However, mutation of these sites in full-length Smo^SD^ reduced but did not eliminate Gprk2-dependent phosphorylation (Figure S2), indicating that additional sites exist.

To look at Smo phosphorylation more directly, we used a label-free quantitative liquid chromatography/tandem mass spectrometry (LC-MS/MS) approach to compare the relative levels of phosphorylated:non-phosphorylated forms of individual Smo phosphopeptides in control and *gprk2* dsRNA-treated cells (see Materials and Methods) [21]. Consistent with the results from the Smo truncations, we detected phosphorylation at all four Ser/Thr residues in cluster 1 (Ser^604^, Thr^606^, Thr^610^, Thr^612^), and at three of five sites (Ser^658^, Ser^659^, Ser^660^) in cluster 3 (Table 1; more detailed quantification included in Table S1). For each phosphospecies we detected, phosphorylation was markedly lower (generally ∼10-fold or more) following *gprk2* depletion. We were unable to obtain peptide coverage in the region of cluster 2. However, we note that others have shown four of the six Ser/Thr residues in this cluster (Ser^626^, Ser^627^, Thr^629^, Ser^633^) are phosphorylated in response to Hh [6].

**Table 1:** Fold downregulation of Smo^SD^ peptide phosphorylation in Gprk2-depleted cells.

Region (amino acids)	Phosphosite	T+AN1	T+AN2	T	C
cluster 1 (604–612)	**S**ITLY/NTHT	*nd*	*nd*	*nd*	10.0
	SI**T**LY/NTHT	*nd*	*nd*	*nd*	*nd*
	**S**I**T**LY/NTHT	*nd*	*nd*	*nd*	6.0
	SITLY/N**T**HT	*nd*	*nd*	*nd*	88.2
	SITLY/NTH**T**	*nd*	*nd*	*nd*	23.9
	SITLY/N**T**H**T**	*nd*	*nd*	*nd*	51.9
cluster 2 (626–635)	SSETNDISST	*nd*	*nd*	*nd*	*nd*
cluster 3 (651–660)	TGAATGN**S**SS TGAATGNS**S**S TGAATGNSS**S**	6.8	8.9	11.7	*nd*
	TGAATGN**SS**S TGAATGN**S**S**S** TGAATGNS**SS**	11	16.4	13.0	*nd*
	TGAATGN**SSS**	12.2	8.8	*nd*	*nd*
cluster 4 (675–683)	**S**VRHVSVES	*nd*	*nd*	4.7	*nd*
	SVRHV**S**VES	7.4	3.2	11.7	*nd*
	**S**VRHV**S**VES	**	**	26.9	*nd*
	SVRHV**S**VE**S**	34.2	7.1	12.2	*nd*
	**S**VRHV**S**VE**S**	*nd*	*nd*	28.0	*nd*
GPS1 (738–745)	RES**S**TSVE	0.9	1.2	1.9	2.0
	RESS**T**SVE	2.4	1.6	1.3	2.0
	RES**ST**SVE	5	2.4	3.2	4.7

**, signal in Gprk2-depleted cells too low to measure. T, trypsin; AN, AspN; C, chymotrypsin.

Phosphorylation at a fourth cluster of three Ser/Thr residues (Ser^675^, Ser^679^, Ser^682^) located in the SAID (Figure 1A) was also much lower in Gprk2-depleted cells (Table 1). Mutation of these sites to Ala in the Smo^SD.c1-3A^ background resulted in a protein (Smo^SD.c1-4A^-GFP) that no longer shifted in response to *gprk2* depletion (Figure 1E) confirming that the four mapped phosphorylation clusters account for all or most Gprk2 phosphorylation. In contrast, we consistently observed comparatively small changes in phosphorylation at GPS1 (Ser^741^ and Thr^742^) in *gprk2*-depleted cells (Table 1). This suggests that another kinase is responsible for a substantial fraction of the phosphorylation at these sites *in vivo*.

As a validation of the LC-MS/MS results, we generated phosphospecific antisera to cluster 1 sites. In Western blots, both anti-Smo-pSer^604^ and anti-Smo-pThr^610^/pThr^612^ antisera recognized wild-type Smo-GFP in extracts from Hh-stimulated cells (Figure 1F and G, lane 1), as well as Smo^SD^-GFP (Figure 1F and G, lane 4). The signals were abolished when the cluster 1 sites were mutated to Ala (Figure 1F and G, lanes 3 and 6), indicating that the antisera specifically recognize the appropriate sites. *gprk2* depletion strongly reduced reactivity of both antisera with Smo (Figure 1F and G, lanes 2 and 5), confirming that the cluster 1 sites are *bona fide* Gprk2 phosphorylation sites.

### Multisite phosphorylation by Gprk2 at clusters 1 and 2 promotes Smo activity

To assess their functional importance, we tested the effects of mutating the Gprk2 phosphosites using *ptc-luciferase (ptc-luc)* transcriptional reporter assays [22] in S2-R+ cells. In control experiments, *gprk2* depletion using a mix of dsRNAs targeting the *gprk2* 5′- and 3′-untranslated regions (UTRs) significantly reduced both Smo phosphorylation and *ptc-luc* reporter activity in Smo^SD^-GFP transfected cells (Figure S3A and B). Both effects could be rescued by expressing a wild-type *gprk2* transgene lacking the UTRs, but not catalytically inactive mutants (Figure S3A and B). *gprk2* depletion had a similar effect on Hh-dependent signaling by endogenous Smo (Figure S3C). Thus the effects of Gprk2 on Smo activity in S2 cells accurately reflect what is observed *in vivo* [18].

**Figure 2:**
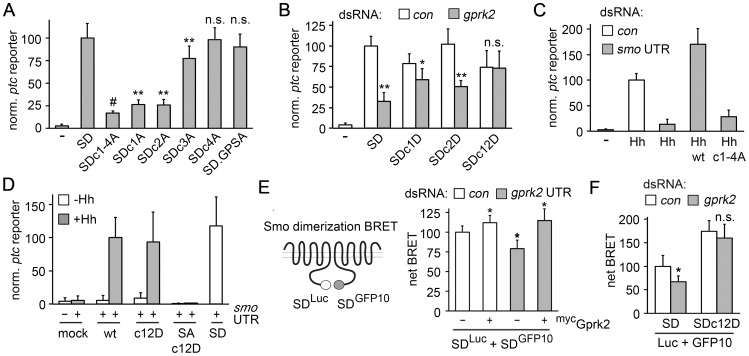
Gprk2 promotes Smo dimerization and activity. (*A*) *ptc-luc r*eporter assay of Gprk2 phosphosite Ala mutants. S2 cells were transfected with the indicated Smo^SD^-GFP variants and *ptc-luc* activity (normalized to *pCMV-renilla*) was measured. Data represent mean ± standard deviation. Ala substitutions at phosphorylation clusters 1 and 2 most strongly impair Smo activity. **, significantly lower than Smo^SD^, *p*<.001. #, significantly lower than Smo^SDc1A^ and Smo^SDc2A^, *p*<.01. n.s., not significantly different from Smo^SD^. (*B*) *ptc-luc* reporter assay of cells treated with dsRNA targeting *β-gal* (control) or *gprk2* and expressing the indicated Smo^SD^-GFP phosphosite Asp variants. The Gprk2 cluster 1 and 2 phosphomimetic form of Smo^SD^ no longer responds to depletion of the kinase. * and **, significantly lower than the respective *β-gal* dsRNA control, *p*<.01 and .001, respectively. n.s., not significantly different from control. (*C*) *ptc-luc* reporter assay of control or *smo 3′UTR* dsRNA-treated cells, transfected without (−) or with a Hh^N^ expression vector along with the indicated Smo-GFP variants. Ala substitution of all four Gprk2 phosphorylation clusters impairs Smo activity. (*D*) *ptc-luc* reporter assay of cells treated with *smo 3′UTR* dsRNA and transfected with or without a Hh^N^ expression vector, along with empty vector (mock) or the indicated Smo-GFP, Smo^SA^-GFP, or Smo^SD^-GFP variants. The Gprk2 phosphomimetic form of Smo does not show constitutive activity. (*E*) BRET efficiency between C-terminally GFP10- and RLucII-tagged Smo^SD^ variants in S2 cells. Cells were treated with control or *gprk2* dsRNA and transfected without (−) or with (+) myc-tagged Gprk2 in addition to the Smo variants. Data are expressed as mean net BRET ± standard deviation. Gprk2 promotes Smo dimerization. *, significantly different from control condition, *p*<.001. (*F*) BRET efficiency between C-terminally GFP10- and RLucII-tagged Smo^SD^ or Smo^SD.c12D^ in S2 cells. Cells were treated with dsRNA targeting *β-gal* (control) or *gprk2* prior to transfection of the indicated Smo variants. The Gprk2 phosphomimetic form of Smo does not respond to *gprk2* depletion. *, significantly lower than control, *p*<.01. n.s., not significantly different from control.

Mutation of all four Gprk2 phosphorylation clusters in the Smo^SD^ background to Ala (Smo^SD.c1-4A^-GFP) reduced *ptc-luc* reporter activity by 80% compared to Smo^SD^-GFP, but did not eliminate it (Figure 2A). Mutation of the four cluster 1 (Smo^SD.c1A^) or six cluster 2 (Smo^SD.c2A^) sites reduced Smo^SD^-GFP-driven *ptc-luc* reporter transcription, but both were significantly more active than Smo^SD.c1-4A^-GFP (Figure 2A). Mutation of the five cluster 3 sites (Smo^SD.c3A^) had less effect, while mutation of the three sites in cluster 4 (Smo^SD.c4A^) had no significant effect on activity (Figure 2A). Consistent with our observations *in vivo*, mutation of the GPS1 and 2 sites also had no significant effect on Smo activity (Figure 2A). Steady-state levels of the various Gprk2 phosphorylation site Smo mutants were similar (Figure S4A). We conclude that phosphorylation at clusters 1 and 2, and to a lesser extent at cluster 3, is required for full Smo activation.

In tests of Smo^SD^-GFP transgenes bearing mutations in only a subset of sites within cluster 1 or 2, we found that no subset impaired activity as much as mutating all sites within a cluster (Figure S5A). We conclude that phosphorylation at many sites rather than a critical activating residue is important for Smo activity. The partial effect on activity when only some sites are mutated suggests that Gprk2 phosphorylation affects Smo activity in a graded manner.

Next, we tested if mimicking the charge effect of Gprk2 phosphorylation at Smo clusters 1 and 2 could compensate for the lack of phosphorylation in Gprk2-depleted cells. In control cells, the Asp-substituted phosphomimetic Smo^SD.c12D^-GFP mutant was ∼25% less active than Smo^SD^-GFP (Figure 2B). This may be because substituting Asp introduces less negative charge than does Ser/Thr phosphorylation under physiological conditions [23]. Importantly, the activity of Smo^SD.c12D^-GFP was unaffected by Gprk2 depletion (Figure 2B). This insensitivity to Gprk2 required Asp substitution in both clusters, as each single cluster mutant (Smo^SD.c1D^ and Smo^SD.c2D^) showed only a partial resistance to depletion of the kinase (Figure 2B). Thus mimicking phosphorylation of Smo^SD^ by Gprk2 at clusters 1 and 2 circumvents the requirement for the kinase itself.

To confirm that the effects on Smo^SD^ activity we observed reflect the normal situation, we mutated the Gprk2 phosphosites in a wild-type Smo background. To minimize any complication arising from endogenous Smo activity, we depleted it by treating the cells with *smo* 3′-UTR dsRNA. Smo depletion was efficient, reducing Hh-stimulated *ptc-luc* reporter activity by 86% (Figure 2C). As expected, re-expressing a wild-type *smo-gfp* transgene made insensitive to the dsRNA by removing the 3′-UTR (Smo^WT^-GFP) restored Hh responsiveness (Figure 2C). Mutation of all four Gprk2 phosphorylation clusters to Ala (Smo^c1-4A^-GFP) strongly impaired this rescue (Figure 2C). Analysis of single cluster mutants confirmed that this was mainly attributable to mutation of clusters 1 and 2 (Figure S5B). In cell surface biotinylation assays, we did not detect any effect of Gprk2 phosphosite mutations on the ability of Smo to traffic to the plasma membrane in response to Hh (Figure S4B). We conclude that wild-type Smo shows a similar dependence on Gprk2 phosphorylation for full activity as Smo^SD^. Unlike PKA and CKI phosphorylation [5], Gprk2 phosphorylation was not sufficient to constitutively activate Smo, as the activity of Smo^c12D^-GFP was as dependent on Hh as Smo^WT^-GFP (Figure 2D). It was also not sufficient to enable Hh-dependent activation of Smo that has all three PKA sites in the SAID mutated to Ala (in Smo^SA.c12D^) (Figure 2D). These results suggest that Gprk2 acts downstream of PKA to enhance Smo activity in Hh-responding cells, consistent with the idea that GRKs preferentially phosphorylate GPCRs in their activated state [24].

### Direct phosphorylation by Gprk2 promotes Smo dimerization

Phosphorylation by PKA and CKI triggers dimerization of Smo C-terminal tails, which promotes high-level signaling activity [1]. To see if direct phosphorylation by Gprk2 affects Smo dimerization, we adapted previously-described biosensors [1] for use in bioluminescence resonance energy transfer (BRET) experiments to measure Smo^SD^ dimerization in intact cells. In control experiments, co-expression of Smo^SD^ molecules C-terminally-tagged with GFP10 (Smo^SD^-GFP10) and *Renilla* luciferase II (Smo^SD^-Luc) yielded a net BRET signal between the two which responded to Gprk2. Gprk2 overexpression, which increased Smo^SD^-GFP phosphorylation (Figure S3B, lanes 1 and 3), also significantly increased net BRET (Figure 2E). Conversely, *gprk2* depletion significantly reduced net BRET (Figure 2E), consistent with a previous report [18]. Re-expressing Gprk2 restored the BRET signal in *gprk2*-depleted cells (Figure 2E), confirming the specificity of the effect of Gprk2 on Smo^SD^ dimerization. Importantly, the cluster 1 and 2 phosphomimetic mutations eliminated the effect of *gprk2* depletion on Smo^SD^ dimerization (Figure 2F). Thus, as in the *ptc-luc* reporter assay, mimicking phosphorylation of Smo^SD^ at clusters 1 and 2 circumvented the requirement for Gprk2. Taken together, the results of our functional studies suggest that Gprk2 directly enhances dimerization of active Smo by phosphorylating it at clusters 1 and 2, driving it into or stabilizing its most active state.

### Loss of direct Gprk2 phosphorylation impairs high threshold Hh target gene expression

**Figure 3:**
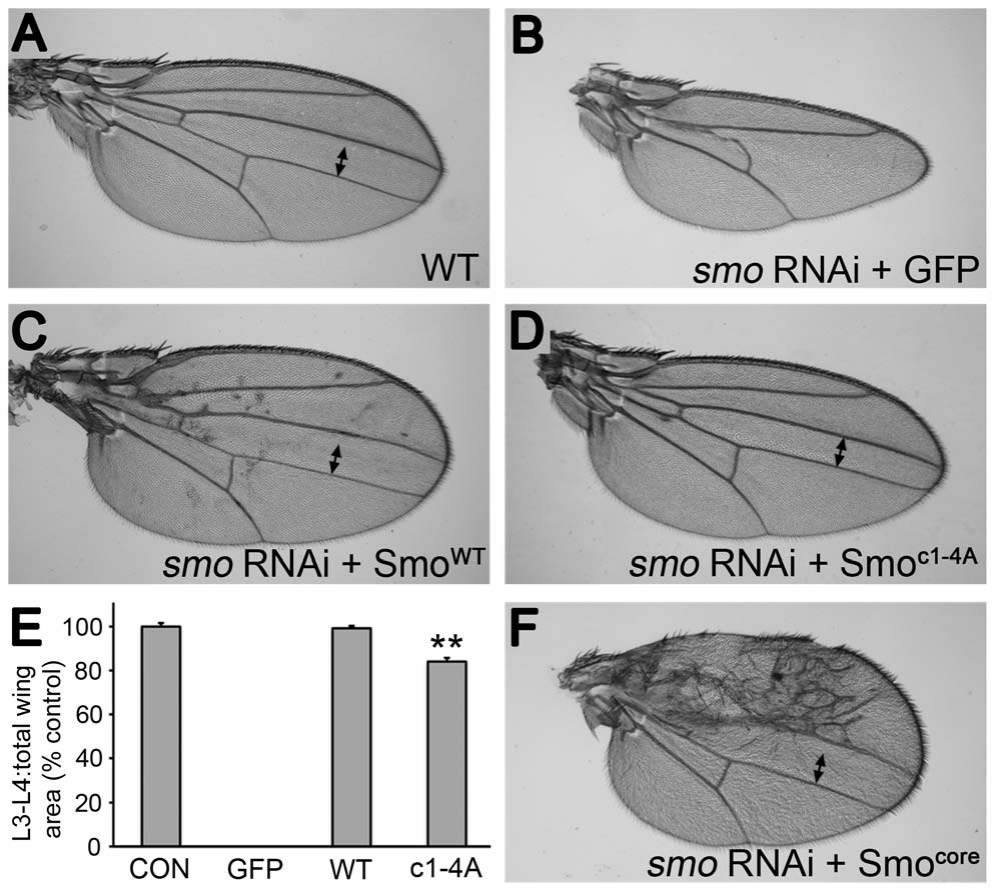
Smo^c1-4A^ substantially but not fully rescues development of wings depleted of endogenous Smo. (A) Wild-type wing. (B) Depletion of endogenous Smo from the entire wing by *nub-GAL4* driven expression of a *smo 3′-UTR* dsRNA transgene (along with GFP as a negative control) led to loss of the central region of the wing patterned by Hh. (C) Reintroduction of wild-type Smo^WT^-GFP expression rescued the central region of the wing and gave a slight Hh gain-of-function phenotype (anterior compartment overgrowth, ectopic vein defects). (D) Reintroduction of Smo^c1-4A^-GFP expression largely rescued the central region of the wing. However, the space between veins 3 and 4 (indicated by two-headed arrow) remained narrower than in controls, indicating that signaling was lower than normal. (E) Measurements of the area bounded by veins 3 and 4 as a proportion of total wing area for the indicated genotypes. Data represent mean ± standard deviation for 5 wings of each genotype. **, significantly lower than Smo^WT^-rescued wings, *p*<.001. (F) Reintroduction of Smo^core^-GFP expression rescued the central region of the wing as efficiently as Smo^WT^-GFP, and induced substantially stronger anterior Hh gain-of-function phenotypes.

*gprk2* mutant animals display several defects, including ectopic accumulation of Smo in Hh-responding cells and strong impairment or loss of intermediate/high but not low threshold target gene expression in the developing wing imaginal disc. To determine to what extent loss of direct phosphorylation of Smo by Gprk2 contributes to these defects, we expressed the wild-type and mutant forms of the protein during wing development. All transgenes were recombined into the same site in the genome using the *Φ*C31-based integration system [25] to ensure equal mRNA expression. For analysis of mutations in the Smo^WT^ background, we co-expressed a *smo* 3′-UTR dsRNA transgene to deplete endogenous Smo. Expression of this dsRNA (together with GFP as a negative control) throughout the developing wing disc using the *nub*-GAL4 driver strongly suppressed Hh signaling, leading to loss of the central region of the adult wing patterned by Hh (Figure 3A and B). Reintroduction of Smo^WT^-GFP fully rescued wing development and induced mild perturbations of anterior patterning indicative of Hh gain-of-function (Figure 3C and E). Smo^c1-4A^-GFP had less activity, with no sign of gain-of-function phenotypes (Figure 3D). However, it rescued development of the central region of the wing, restoring growth to about 84% of the normal size (Figure 3D and E), suggesting that loss of direct Gprk2 phosphorylation has relatively mild effects on Smo activity *in vivo*.

**Figure 4:**
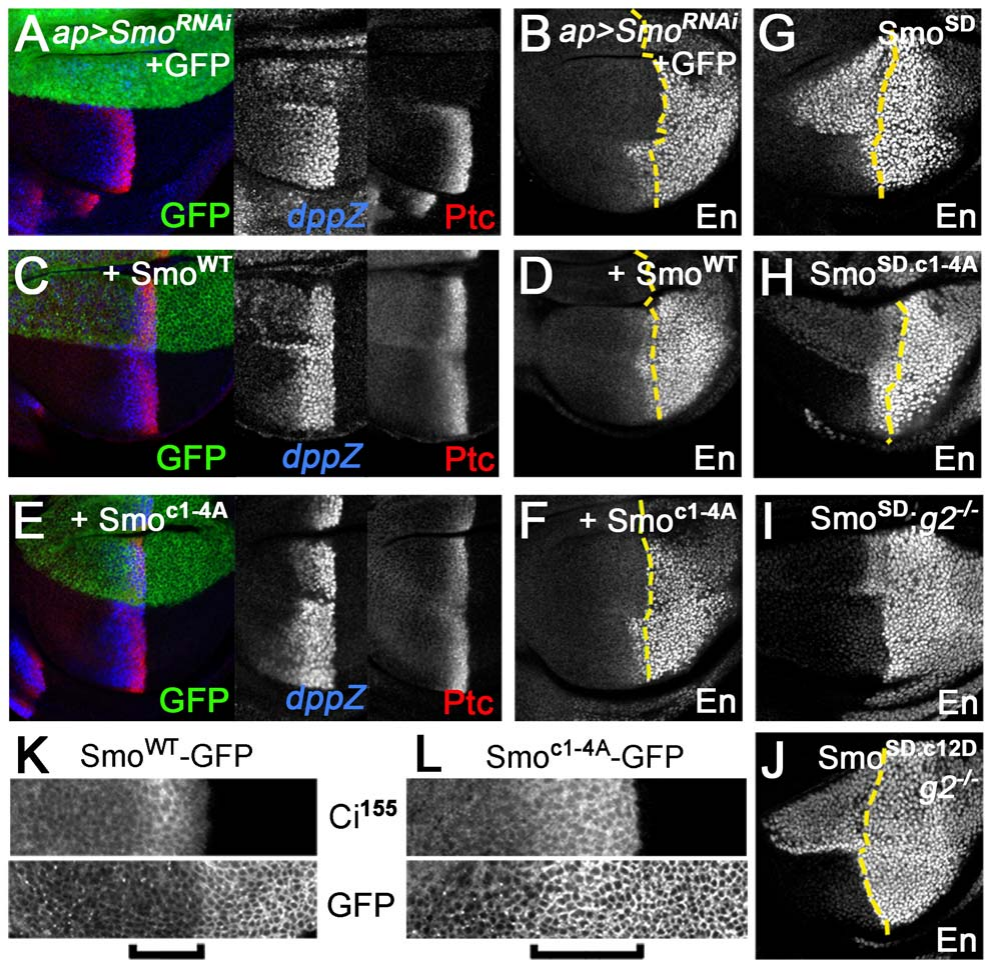
Gprk2 phosphorylation is required for maximal Smo activity *in vivo*. Confocal micrographs of wing discs, oriented with anterior [A] compartment to left, dorsal [D] compartment up. (*A*–*F*) Endogenous Smo was depleted from the D compartment by *ap-GAL4*-driven expression of *smo 3′UTR* dsRNA and replaced with GFP (*A* and *B*), Smo^WT^-GFP (*C* and *D*), or Smo^c1-4A^-GFP (*E* and *F*). Immunostaining: Ptc and *dpp-LacZ* (*A*, *C*, and *E*); En (*B*, *D*, and *F*). *Dotted lines*: anterior/posterior (A/P) compartment boundary (based on Ci immunostaining, not shown). Smo^c1-4A^ rescued Dpp and Ptc but not En expression. (*G*–*J*) En expression in wing discs expressing Smo variants in D compartment: Smo^SD^ (*G* and *I*), Smo^SD.c1-4A^ (*H*), or Smo^SD.c12D^ (*J*). Discs are wild-type (*G* and *H*) or *gprk2* mutant (*I* and *J*) background. *Dotted lines*: A/P compartment boundaries. Ala substitution of all four Gprk2 phosphorylation clusters modestly impairs Smo^SD^ activity *in vivo*, whereas phosphomimetic substitutions restore full Smo^SD^ activity in the absence of Gprk2. (*K–L*) GFP fluorescence in D compartment of wing discs with endogenous Smo depleted and replaced with Smo^WT^-GFP (K) or Smo^c1-4A^-GFP (*L*). *Brackets*: Hh-responding cells (identified by increased Ci^155^ immunostaining). The Gprk2 non-phosphorylatable form of Smo accumulates ectopically in Hh-responding cells.

Analysis of Hh target gene expression in wing discs led us to a similar conclusion. Expression of *smo* 3′-UTR dsRNA in the dorsal compartment of the disc using *ap*-GAL4 eliminated expression of the intermediate threshold target gene *ptc* and high threshold target anterior *en*, and strongly reduced expression of the low threshold target *dpp* (Figure 4A and B). Reintroducing Smo^WT^-GFP in this background restored Hh-dependent expression of all three genes, with signs of weak ectopic *dpp* and *ptc* expression apparent in far anterior cells (Figure 4C and D). In contrast, Smo^c1-4A^-GFP rescued low (*dpp*) but not high (*en*) threshold target gene expression (Figure 4E and F), matching observations in *gprk2* mutants [16]–[18]. Hh-dependent expression of *ptc* was partially rescued (Figure 4E). However, Ptc levels in Smo^c1-4A^-GFP-expressing dorsal cells were higher than is typically seen in *gprk2* mutants, consistent with the observation that reduced levels of cAMP and PKA also contribute to the impairment of Hh signaling activity in the absence of Gprk2 [19].

The Gprk2 phosphosite mutations had a similar partial effect on the activity of Smo^SD^. Hh-independent ectopic expression of anterior *en* [5] was substantially lower in Smo^SD.c1-4A^-GFP-expressing discs than in those expressing Smo^SD^-GFP, but was still readily detectable (Figure 4G and H). As in S2 cells, mimicking Gprk2 phosphorylation restored the ability of Smo^SD^ to drive strong ectopic *en* expression in *gprk2* mutant discs (Figure 4I and J). Taken together, our *in vivo* analyses support the conclusion that direct phosphorylation by Gprk2 is not essential for Smo activity, but enhances it to its highest level.

Interestingly, the cluster 1–4 Ala-substituted form of Smo faithfully phenocopied the effects of Gprk2 loss on Smo accumulation. In discs depleted of endogenous Smo, wild-type Smo-GFP accumulated in the normal pattern [26], including low levels in most anterior Hh-responding cells (identified by high levels of stabilized Ci) (Figure 4K, *bracket*). In contrast, Smo^c1-4A^-GFP accumulated ectopically in Hh-responding cells (Figure 4L, *bracket*), as endogenous Smo does in *gprk2* mutants [16], [18]. We conclude that Gprk2 phosphorylation is required for the downregulation of Smo seen in cells where the Hh signaling pathway is active.

### An evolutionarily-conserved mechanism for Smo regulation and signaling

**Figure 5:**
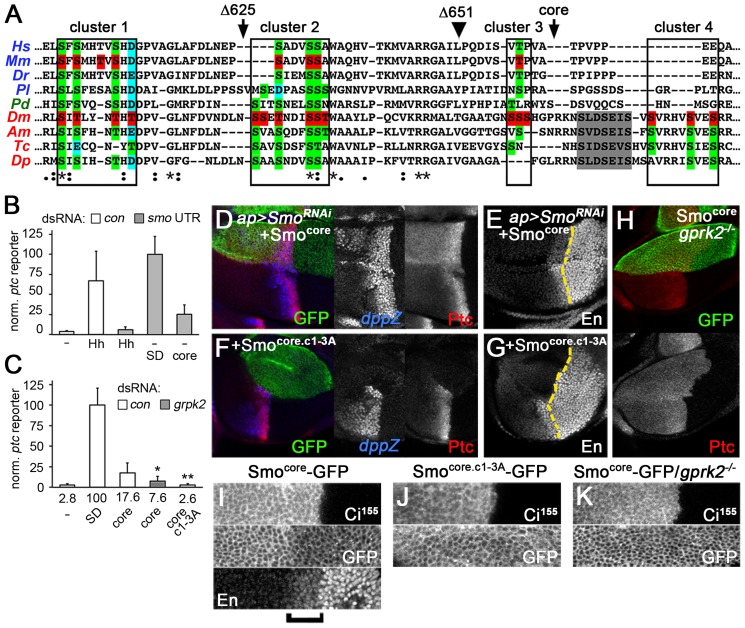
The conserved core of Smo is a GRK-regulated, signaling-competent protein. (*A*) Sequence alignment of a portion of the Smo C-terminus. Orthologues from each of the three main bilaterian branches are included: deuterostomes - human (Hs), mouse (Mm), zebrafish (Dr), sea urchin (Pl) [*in blue*]; lochotrophozoans - *Platynereis* (Pd) [*in green*]; and ecdysozoans - *Drosophila* (Dm), honey bee (Am), red flour beetle (Tc), and water flea (Dp) [*in red*]. *Boxes*: Gprk2 phosphorylation clusters. *Arrowhead*: approximate end of broad sequence conservation, corresponding to amino acid 651 in *Drosophila* Smo. *Grey shading*: first PKA/CKI phosphorylation cluster in the SAID. *Red highlighting*: mapped phosphorylation sites in mouse and *Drosophila* Smo. *Green highlighting*: conserved Ser/Thr residues. *Blue highlighting*: Asp/Glu residues where negative charge is conserved. *Arrows*: sites of truncation in Smo^Δ625^ and Smo^core^ proteins. (*B*) *ptc-luc* reporter assay of cells treated with control or *smo 3′UTR* dsRNA and transfected with empty vector (−) or expression vectors for Hh^N^, Smo^SD^-GFP, or Smo^core^-GFP. Smo^core^-GFP was active in endogenous Smo-depleted cells. (*C*) *ptc-luc* reporter assay of cells treated with control or *gprk2* dsRNA and transfected with empty vector (−), Smo^SD^-GFP, Smo^core^-GFP, or Smo^core.c1-3A^-GFP. Fold-induction of the reporter in each condition is shown. Smo^core^-GFP signals in a Gprk2 phosphorylation-dependent manner. * and **, significantly lower than Smo^core^, *p<0*.05 and 0.001, respectively. (*D–G*) Wing discs with endogenous Smo depleted in the dorsal compartment and replaced with Smo^core^-GFP (*D* and *E*) or Smo^core.c1-3A^-GFP (*F* and *G*), immunostained for Ptc and *dpp-LacZ* (*D* and *F*) or En (*E* and *G*). Smo^core^-GFP rescued Dpp, Ptc, and even En expression, dependent upon Gprk2 phosporylation. (*H*) *gprk2* mutant wing disc with Smo^core^-GFP expressed in the D compartment, immunostained for Ptc. Smo^core^-GFP activity required Gprk2. (*I–K*) GFP fluorescence in D compartment of wing discs with endogenous Smo depleted and replaced with Smo^core^-GFP (*I* and *K*) or Smo^core.c1-3A^-GFP (*J*). Discs are wild-type (*I* and *J*) or *gprk2* mutant (*K*) background. The A/P boundary was identified by Ci^155^ immunostaining. *Bracket*: Cells responding strongly to Hh (identified by increased En immunostaining).

Broad sequence conservation in the cytoplasmic C-terminus of Smo is limited to the membrane-proximal 100 amino acids (up to amino acid 651 in *Drosophila* Smo - see Figure 5A). The remaining 385 amino acids of *Drosophila* Smo, including the SAID, PKA/CKI sites, and binding sites for the kinesin-like protein Cos2 [8], [9] are only conserved among arthropods. The absence of PKA sites in vertebrate Smo orthologues was recently explained with the identification of GRK2 and CKI as the activating kinases for these proteins. GRK2 and CKI phosphorylate twelve sites in the cytoplasmic tail of mSmo, inducing a change to an active conformation, possibly by neutralizing a nearby stretch of basic amino acids as in flies [1], [2]. Most of these sites are conserved in vertebrate Smo orthologues, suggesting that PKA and CKI together replace the function of PKA in activating vertebrate Smo through a mechanism that is similar to, but molecularly distinct from, that in flies [2].

In broader sequence alignments that include Smo orthologues from each of the three main bilaterian clades, we noted that the Gprk2 phosphorylation sites we mapped in *Drosophila* Smo overlap with the mSmo phosphorylation sites and show a remarkable degree of conservation in other species (Figure 5A). In total, eight of the sites in Gprk2 clusters 1–3 correspond to phosphorylation sites in mSmo that are nearly universally conserved throughout the bilaterians, either as Ser/Thr residues or as Asp/Glu residues (consistent with the evolution of phosphosites from functionally similar negatively charged residues [23]). The first two clusters (PS0 and PS1) in mSmo, which contain most of the conserved sites, were functionally the most important [2], as are the corresponding cluster 1 and 2 sites in *Drosophila*. Thus both at the level of sequence and function, the Gprk2/GRK2/CKI phosphorylation sites appear to represent an evolutionarily ancient and conserved mechanism of Smo regulation.

If they share a common regulatory mechanism, we hypothesized that the bilaterian Smo orthologues may also have retained the activity of the ancestral form of Smo that was being regulated, i.e. they may share a conserved molecular signaling mechanism. To address this, we tested the effects of expressing just the highly conserved portion of Smo (Smo^core^ - amino acids 1–663, truncated just after the broadly conserved third Gprk2 phosphorylation cluster; Figure 5A) on Hh target gene expression. Consistent with our hypothesis, we found that Smo^core^ was capable of activating *ptc-luc* reporter expression in endogenous Smo-depleted cells, to about 25% the level of Smo^SD^ (Figure 5B). This constitutive activity of Smo^core^ was strongly reduced or abolished by *gprk2* depletion or mutation of the Gprk2 phosphorylation sites (Smo^core.c1-3A^), respectively (Figure 5C), indicating that Smo^core^-GFP activity is regulated by Gprk2 phosphorylation. In endogenous Smo-depleted wing discs, Smo^core^-GFP rescued Hh-dependent expression of *dpp*, *ptc*, and even *en* (though *en* levels remained lower than wild-type) (Figure 5D and E), as well as overall wing development (Figure 3F). It also drove ectopic expression of *dpp* and *ptc* (Figure 5D), producing anterior Hh gain-of-function phenotypes in adult wings that were even stronger than those observed with Smo^WT^-GFP (Figure 3F). Consistent with the results of *ptc-luc* reporter assays, Smo^core.c1-3A^-GFP displayed no ability to rescue target gene expression in endogenous Smo-depleted discs (Figure 5F and G). Similarly, when expressed in *gprk2* mutant discs, the ability of Smo^core^-GFP to activate *ptc* expression was lost, and it appeared to inhibit the residual *ptc* expression resulting from endogenous Smo activity (Figure 5H). Thus, as in the S2 cell assays, signaling *in vivo* was dependent on phosphorylation by Gprk2. We conclude that the highly conserved core of Smo is a GRK-regulated protein that contains sequences sufficient for activating downstream signaling.

Interestingly, deleting the C-terminus altered the pattern of Smo accumulation in discs in a manner that was inversely correlated with its activity. Normally, Smo levels are low in the far A compartment, where Smo is inhibited by the activity of Ptc, and high in the P compartment and first few rows of A Hh-responding cells, where Smo activity is high because Ptc is either not expressed (P compartment) or strongly inhibited by Hh (e.g. Figure 4K). In contrast, Smo^core^-GFP levels were highest in far A cells where Smo^core^ activity is lowest, and low throughout the Hh-responsive A zone and P compartment, where Smo^core^ activity is expected to be highest (Figure 5I). This pattern is reminiscent of the regulation of many GPCRs, which undergo GRK phosphorylation-dependent internalization and, in many cases degradation, after being activated [27]. Removing Gprk2 or mutating its phosphorylation sites eliminated the downregulation of Smo^core^ in cells where the Hh pathway is strongly activated (Figure 5J and K), indicating that the effect is indeed the result of direct phosphorylation. Thus Gprk2 controls both the accumulation and activity of Smo^core^, as GRK2 does ciliary accumulation and activity of mSmo [2].

**Figure 6:**
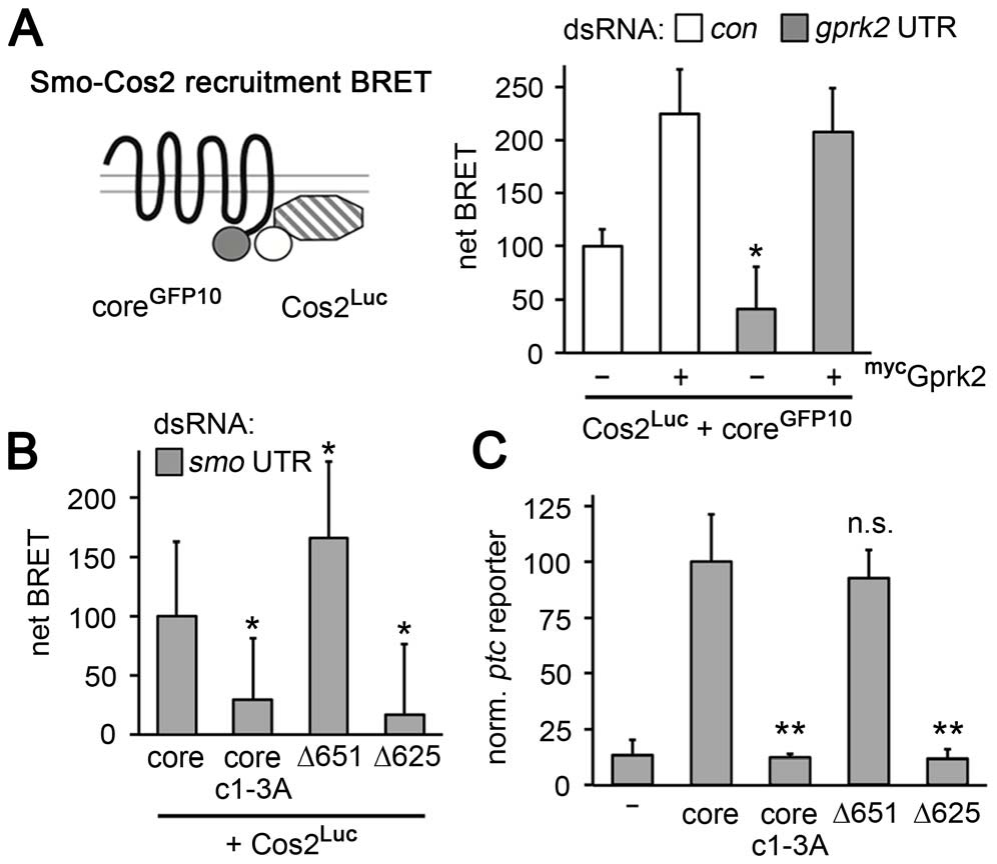
Smo^core^ recruits Cos2. (*A*) BRET efficiency between C-terminally GFP10-tagged Smo^core^ and C-terminally RLucII-tagged Cos2 in S2 cells. Cells were treated with *β-gal* (control) or *gprk2* dsRNA and transfected without (−) or with (+) myc-tagged Gprk2 in addition to Smo^core^. Data are expressed as mean net BRET ± standard deviation. Gprk2 promotes interaction between Smo^core^-GFP10 and Cos2-Luc. *, significantly lower than *β-gal* dsRNA-treated control cells, *p*<.01. (*B*) BRET efficiency between C-terminally GFP10-tagged truncated Smo variants and RLucII-tagged Cos2 in S2 cells treated with *smo 3′UTR* dsRNA to deplete endogenous Smo. Interaction with Cos2 depended upon Gprk2 phosphorylation and Smo sequences between amino acids 625–651. *, significantly different than Smo^core^-GFP10, *p*<.01. (*C*) *ptc-luc* reporter assay of cells expressing truncated Smo variants. The ability to stimulate reporter expression depended upon Gprk2 phosphorylation and Smo sequences between amino acids 625–651. **, significantly lower than Smo^core^-GFP, *p*<.001.

In *Drosophila* and vertebrates, Smo binds to Cos2 and its orthologue Kif7, respectively [8]–[10], and these proteins are required for full activation of the pathway [11]–[13]. To see if Smo^core^ recruits Cos2, we adapted previously-described FRET biosensors [14] to measure Cos2-Smo^core^ interaction by BRET. Co-expression of Smo^core^ C-terminally-tagged with GFP10 (Smo^core^-GFP10) and Cos2 C-terminally tagged with *Renilla* luciferase II (Cos2-Luc) yielded a net BRET signal that was responsive to Gprk2. Gprk2 overexpression increased Smo^core^-Cos2 BRET more than 2.2-fold, whereas *gprk2* depletion reduced it by 60% (Figure 6A). The effects were specific, as re-expressing Gprk2 in *gprk2*-depleted cells fully rescued Smo^core^-Cos2 BRET (Figure 6A). Smo^core.c1-3A^ mimicked the effect of *gprk2* depletion on Smo^core^-Cos2 interaction, even after depletion of endogenous Smo (ruling out the possibility that the BRET signal is due to interaction of Cos2-Luc with endogenous Smo in Smo/Smo^core^ oligomers) (Figure 6B). These results suggest that Smo^core^ recruits Cos2, in a Gprk2 phosphorylation-dependent manner.

Previous studies localized separate binding sites for Cos2 in the Smo C-terminus between amino acids 651–686 and 818–1035 [8], [9], most of which is missing in Smo^core^. To see if the Cos2 recruitment activity we observed was due to binding between amino acid 651 and the end of Smo^core^ at amino acid 663, we made a further truncation to amino acid 651 (see Figure 5A). Smo^Δ651^ interacted with Cos2 in the BRET assay even more efficiently than Smo^core^, in endogenous Smo-depleted cells (Figure 6B), indicating that recruitment was not to the previously mapped sites. Further truncation to amino acid 625 (Smo^Δ625^), just N-proximal to Gprk2 phosphorylation cluster 2 (Figure 5A), eliminated Cos2 recruitment (Figure 6B). Importantly, the ability of these truncated Smo proteins to recruit Cos2 correlated with their activity in *ptc-luc* reporter assays (Figure 6C). We confirmed that all proteins were expressed at similar levels (Figure S6). These results suggest that the region between 625 and 651 contains a novel Cos2 binding site that positively transduces signals downstream of Smo. Unlike previously mapped sites, this one is located in a region that is broadly conserved among bilaterian species.

## Discussion

Previous studies of GRK phosphorylation of *Drosophila* and mouse Smo proteins identified sites that were not conserved between the two [2], [18], consistent with the high degree of sequence divergence in much of the cytoplasmic C-terminus. In fact, a large body of data concerning kinase regulation of Smo proteins and their engagement of the downstream signaling apparatus have supported the view that, although they ultimately do similar things, fly and vertebrate proteins must do so through distinct molecular mechanisms [3], [28]. In this study, we characterized the effects of direct Gprk2 phosphorylation on Smo in *Drosophila* by mapping four new clusters of Gprk2 phosphorylation sites, two of which are situated in the highly-conserved portion of the cytoplasmic C-terminus. We find that direct phosphorylation by Gprk2 is not strictly required for Smo activation, but acts to enhance Smo dimerization and signaling activity in the Hh response. Furthermore, we demonstrate that the highly conserved core of Smo contains sequences sufficient to recruit Cos2 and activate downstream signaling, in a GRK phosphorylation-dependent manner. These results suggest that GRK phosphorylation in the membrane proximal C-terminus is an evolutionarily ancient mechanism of Smo regulation, and point to a higher degree of functional similarity at a molecular level among bilaterian Smo orthologues than was previously recognized.

### Multisite phosphorylation of Smo by Gprk2

GRKs tend to phosphorylate multiple Ser/Thr residues within short stretches of amino acids in their GPCR substrates [29]–[32]. We identified 18 Ser/Thr residues in four such clusters in the Smo C-terminus, mutation of which abolished Gprk2-dependent phosphorylation. Of these, we confirmed by LC-MS/MS analysis that 10 sites (Ser^604^, Thr^606^, Thr^610^, Thr^612^, Ser^658^, Ser^659^, Ser^660^, Ser^675^, Ser^679^, Ser^682^) are phosphorylated by Gprk2, with Ser^604^, Thr^610^, and Thr^612^ being further validated using phosphospecific antisera. Two of the sites in cluster 3 (Thr^551^ and Thr^555^) are likely not Gprk2 targets, as we did not detect phosphorylation at either site in control cells. We did not obtain peptide coverage in the region containing the remaining six sites (Ser^626^, Ser^627^, Thr^629^, Ser^633^, Ser^634^, and Ser^635^), which make up cluster 2; however, phosphorylation at four of these sites has been observed by others [6]. Our results suggest that Gprk2 does phosphorylate at least some of these residues, since phosphomimetic mutations in cluster 2 are required to fully rescue Smo activity in *gprk2*-depleted cells. In mSmo, CK1 phosphorylates the sites corresponding to cluster 1 whereas GRK2 phosphorylates cluster 2 [2]. The role of CKI does not seem to be conserved, as CKI depletion had no appreciable effect on phosphorylation of the sites we mapped in *Drosophila* Smo (Figure S7). We observed Gprk2-dependent changes in phosphorylation at the GPS1 sites by LC-MS/MS but they were relatively small, suggesting that another kinase also phosphorylates these sites. In total, then, Gprk2 appears to be the principle kinase responsible for phosphorylating between 11 and 16 sites in the *Drosophila* Smo C-terminus.

### Direct phosphorylation by Gprk2 enhances Smo activity and turnover

Gprk2 regulates Smo stability and activity, with both effects mediated at least partly through direct phosphorylation. Several observations indicate that Gprk2 directly enhances Smo activity in Hh-responding cells. The extent of the conformational shift that Smo undergoes upon activation is lower in *gprk2*-depleted cells, indicative of a lower activity state [18]. We confirmed previous observations that its ability to dimerize is also partly compromised [18]. The result is less robust activation of target gene expression. The reduced activity of Smo mutants with Ala substitutions at the Gprk2 phosphorylation sites in *ptc-luc* reporter assays confirms that Gprk2 enhances Smo activity by phosphorylating it. In particular, phosphorylation at clusters 1 and 2 seems to be critical, as Ala substitutions at these clusters caused the strongest impairment of target gene activation whereas phosphomimetic substitutions at both rendered Smo resistant to the effects of *gprk2* depletion, as assessed by dimerization and target gene activation. The latter observations strongly argue against Gprk2 having a catalytic activity-independent function in regulating Smo, as has been suggested [18]. The effects of mutating the Gprk2 phosphorylation sites in Smo were more subtle *in vivo* than in *ptc-luc* reporter assays in S2 cells. We speculate that this is due to the artificial nature of the *ptc-luc* reporter assay itself. Although Smo^SD^ expression or Hh treatment can yield 50-fold or more activation of the reporter, proteins that induce a ∼10-fold increase in these assays (Smo^SD.c1-4A^, Smo^core^) are capable of activating most target gene expression *in vivo*. Activity above this level may simply be non-physiological.

**Figure 7:**
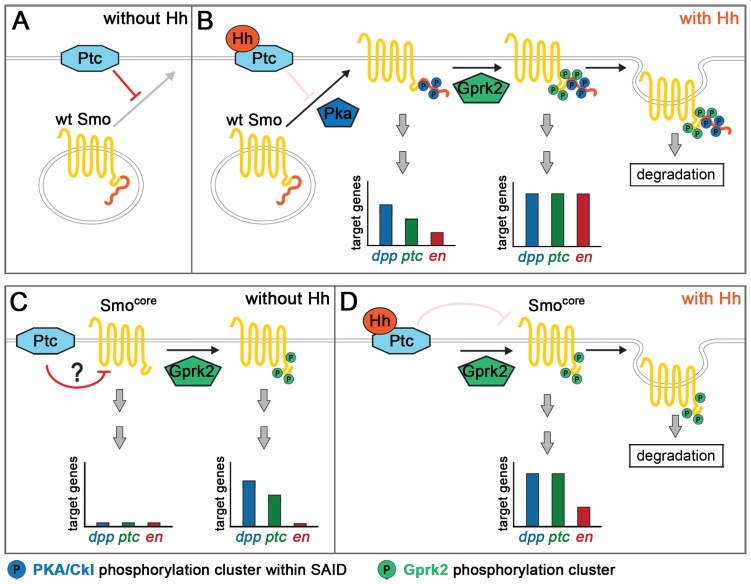
Model for Gprk2 function in Smo/Smo^core^ activation. (A) In the absence of Hh, Ptc inhibits Smo by promoting its ubiquitin-dependent degradation and preventing it from accumulating at the plasma membrane. (B) Binding of Hh inhibits Ptc. Phosphorylation of the SAID by PKA and then CKI (not shown) leads to Smo accumulation at the plasma membrane, and promotes its dimerization and shift to an active conformation. In this state, target gene expression is strongly, but not fully, activated. Gprk2 phosphorylates Smo at the plasma membrane, driving Smo into its most active state and promoting full target gene expression. Gprk2 phosphorylation also promotes internalization and degradation of activated Smo in Hh-responding cells, limiting the duration of Smo signaling. (C) Smo^core^ is partially resistant to downregulation by Ptc, likely because it lacks the inhibitory SAID. Nonetheless, Ptc does inhibit Smo^core^ activity. GRK phosphorylation partially activates Smo^core^ even in the absence of Hh, leading to some constitutive expression of low and intermediate threshold target genes. (D) Binding of Hh inactivates Ptc, relieving it inhibition of Smo^core^. Gprk2-phosphorylated Smo^core^ promotes strong expression of low and intermediate threshold targets and weak expression of high threshold targets. As with full-length Smo, Gprk2 phosphorylation promotes internalization and degradation of Smo^core^ in Hh-responding cells.

Although Gprk2 phosphorylation contributes to activating Smo, it is neither necessary nor sufficient. Smo activation appears to be a two-step process, with phosphorylation by PKA and CKI in the SAID serving as the principal trigger (Figure 7A and B). SAID phosphorylation has at least two effects. First, it inhibits Smo ubiquitination and its subsequent endocytosis and degradation, leading to Smo accumulation at the cell surface [33], [34]. Second, it promotes Smo dimerization and a shift to a more active conformation [1]. In our analysis, mimicking PKA/CKI phosphorylation at all nine sites was sufficient for full expression of all target genes except *en*, which was only partially activated, independent of Gprk2. Thus full PKA/CKI phosphorylation is sufficient to strongly, but not completely, activate Smo. Full activation requires phosphorylation by Gprk2.

Mimicking Gprk2 phosphorylation alone had no effect on Smo activity in the absence of Hh, nor could it activate in the absence of phosphorylation by PKA/CKI. This could be because access to the Gprk2 sites is blocked without prior PKA/CKI phosphorylation. However, it seems more likely that the effect of PKA/CKI in controlling accumulation of Smo at the cell surface, where GRKs are typically localized [35], limits the influence of Gprk2 on Smo. Once Smo accumulates at the cell surface, Gprk2 appears to phosphorylate it constitutively.

PKA and CKI phosphorylation disrupts electrostatic interactions between the SAID domain and the distal C-terminus, thereby promoting a more open and active Smo conformation [1]. Gprk2 phosphorylation appears to act by a different mechanism. The functionally important Gprk2 phosphorylation sites are not located in the SAID domain, and Gprk2 phosphorylation regulates the activity of the truncated Smo^core^ protein that lacks both the SAID domain and distal C-terminus. These observations favour a model in which Gprk2 phosphorylation more directly affects the conformation of the proximal C-terminus or seven transmembrane domain portion of Smo to enhance its activity.

Previous studies have shown that Gprk2 promotes Smo internalization and degradation in response to Hh [16]–[18]. The Smo^c1-4A^ mutant accumulated ectopically in wild-type Hh-responding cells, as endogenous Smo does in *gprk2* mutants, demonstrating that Gprk2 triggers Smo turnover by directly phosphorylating it. This is consistent with the typical role of GRKs in receptor desensitization, and supports the conclusion that Gprk2 phosphorylation limits the duration of Smo signaling [16].

### Broadly conserved Smo regulatory and signaling mechanisms

The striking conservation of the first two Gprk2 phosphorylation clusters in all bilaterian Smo proteins clearly points to an ancient origin and common function. Indeed, there are some parallels between the functions of Gprk2 phosphorylation of *Drosophila* Smo and GRK2/CKI phosphorylation of mSmo [2]. In both cases, it is the same two membrane-proximal clusters that are most important for function. Ala substitution of these sites in either protein impairs dimerization of the C-terminal tail and target gene expression. In both cases, the magnitude of the effect correlates with the number of substitutions, implying that phosphorylation at these sites can activate Smo in a dose-dependent manner. Phosphorylation at these sites also controls trafficking of Smo in Hh-responding cells in both systems, being required for Shh-dependent ciliary translocation of mSmo and for Hh-dependent internalization and downregulation of *Drosophila* Smo.

One important difference is that GRK phosphorylation is required and sufficient for Smo activation in mammals but not flies. In this regard, mSmo behaves more like the truncated Smo^core^ protein (Figure 7C and D). Our analysis indicates that Smo^core^ contains all the sequences necessary for activating downstream signaling, although it may do so less effectively than full-length Smo. Like mSmo, Smo^core^ signaling is strongly or completely inhibited by Ala substitution of Gprk2 clusters 1–3, or by removal of the kinase, indicating that it is strictly dependent upon phosphorylation by Gprk2. Smo^core^ displays some constitutive activity. However, it is also regulated by Ptc. For example, in Smo^core^-GFP-expressing discs, cells that have higher Ptc activity (such as those in the far A) express target genes at lower levels than Hh-responding cells at the A/P boundary. Ptc overexpression in S2 cells reduces Smo^core^-driven *ptc-luc* reporter expression (not shown). Because Ptc downregulates full-length Smo through a mechanism involving ubiquitination of the SAID [36], the absence of this domain in Smo^core^ could explain its accumulation in far A cells. How Ptc regulates Smo^core^ activity is unclear, but could be related to its proposed function in regulating the levels of a Smo agonist or antagonist [37].

Signaling downstream of both *Drosophila* and mammalian Smo proteins involves Cos2/Kif7. Despite lacking previously mapped Cos2 interaction domains, Smo^core^ is capable of recruiting Cos2, and the ability of successive C-terminally truncated forms of Smo^core^ to do so correlates with their ability to stimulate target gene expression. This truncation approach allowed us to identify a region required for Smo-Cos2 interaction between amino acids 625–651. Gprk2 phosphorylation cluster 2 falls within this region, and the ability of Smo^core^ to recruit Cos2 is strongly influenced by Gprk2 phosphorylation. Phosphorylation may influence Smo^core^ conformation in a way that favours Cos2 interaction. Alternatively, Cos2 may interact with this region preferentially in a phosphorylated state, as β-Arrestins do with GRK-phosphorylated GPCRs [38]. As this region falls in the portion of the Smo C-terminus that is broadly conserved, it could represent a mechanism of Smo regulation and signaling that is common to all bilaterian species, something that has previously been lacking.

### Evolutionary origin of Smo proteins

Our analysis of Smo^core^ provides some insights into the potential evolutionary origin of Smo. Smo^core^ is a highly conserved, minimal functional form of Smo, and we speculate that it may closely resemble the ancient form of Smo in the common bilaterian ancestor. It displays a mode of regulation that is typical of GPCR desensitization, suggesting that the ancient form of Smo may have behaved more like a classical GPCR. The evidence suggests that different mechanisms have evolved in different lineages for restricting the activity of Smo^core^, in the form of C-terminal negative regulatory domains. In vertebrates, a stretch of positively charged residues in the C-terminus serves to keep the core in an inactive conformation, and phosphorylation primarily at the first two GRK/CKI clusters overcomes this effect. This same phosphorylation mechanism has been retained in *Drosophila* and other arthropods. However, through evolutionary time, this group appears to have acquired a PKA/CKI-regulated autoinhibitory domain that has come to dominate Smo activity. In contrast to vertebrates, the ancestral GRK mechanism has been relegated to a modulatory role in flies, where it is required to achieve the highest level of Smo signaling. The principal role of driving Smo into an open conformation and activating downstream signaling has been assumed by PKA/CKI phosphorylation of the SAID domain. One consequence of SAID phosphorylation is recruitment of Cos2 and Fu to binding sites in the nonconserved distal C-terminus, leading to Cos2-dependent Fu dimerization and activation [14], [39], [40]. Fu dimerization is sufficient to strongly activate Hh signaling [14]. This Fu-dependent mechanism, mediated via the nonconserved Cos2 and Fu binding sites, is not thought to exist in vertebrate Hh signaling, and may account for the difference in signaling strength between full-length Smo and Smo^core^. Further analysis of Smo^core^ will be necessary for a full understanding of how *Drosophila* Smo connects to the downstream signaling apparatus, and should provide insights into a signaling mechanism common to all Smo proteins.

## Materials and Methods

### Constructs

For expression of Smo mutants, we first silently mutated codons 458 and 459 of wild-type and Smo^SD^ [5] coding sequences to introduce an *Eco*RV site. A 1023 nt *Eco*RV-*Eco*RI fragment of wild-type or Smo^SD^ coding sequence containing codon 458–798 and harboring all Gprk2 and PKA phosphorylation sites was subcloned into *pBluescript* (*pBS*). The resulting constructs were used as templates for multiple rounds of PCR-based site-directed mutagenesis in order to mutate all Gprk2 sites. The modified *Eco*RV-*Eco*RI fragments were then cloned back into full-length Smo expression plasmids. To generate Smo truncations (Smo^core^ - amino acids 1–663, Smo^Δ651^ - amino acids 1–651, Smo^Δ625^ - amino acids 1–625 and Smo^Δ603^ - amino acids 1–603) Smo sequences between the *Eco*RV site at codon 458–459 and the indicated 3′ codon were PCR amplified, introducing a 3′ *Not*I site. The resulting *Eco*RV-*Not*I fragments were cloned into Smo expression vectors. All Smo constructs were C-terminally tagged with either GFP, GFP10 or RLucII. The tags were engineered as a cassette flanked by *Not*I and *Kpn*I restriction sites. Coding fragments were cloned into expression constructs for use in cell culture (*pRmHa3.puro* [26] containing the *metallothionein* promoter) and flies (*pUAST-AttB* [25]). *UAS-smo-3′UTR-dsRNA* was generated by cloning a genomic PCR-generated fragment containing nucleotides 2L:281756..281981 of the *smo* 3′-UTR. The 226 nt long fragment was cloned between the EcoRI-AvrII sites and in the opposite orientation between the NheI-XbaI sites of *pWIZ* (*Drosophila* Genomics Resource Centre). To generate catalytically inactive forms of Gprk2, point mutations changing Lys^338/339^→Met (Gprk2^kd1^ [18]) or Asp^453^→Asn (Gprk2^kd2^) were generated by PCR mutagenesis and cloned downstream of a Myc-epitope tag in *pRmHa3.puro*. A C-terminal luciferase-tagged Cos2 expression construct used in BRET assays was engineered by flanking the Cos2 coding sequence at the 5′ and 3′ end with an *Eco*RI and a *Not*I site, respectively. The resulting *Eco*RI-*Not*I fragment was cloned into *pRmHa3.puro*. The RLucII cassette described above was cloned downstream of the Cos2 sequence at the *Not*I site. Fu coding sequence was cloned downstream of a Myc-epitope tag in *pRmHa3.puro*. Sequences of all constructs were verified. For experiments involving Hh treatment, cells were co-transfected with *pRmHa3.puro/Hh^N^* [26], which encodes an active N-terminal fragment of *Drosophila* Hh. For *ptc-luc* reporter assays a mixture of the following constructs was used: *pRmHa3/Ci* (a gift from S. Cohen, University of Copenhagen, Denmark), *pGL.basic/ptcΔ136-luc* [22], and *pRL/CMV* (Promega).

### Fly strains and reagents

All *UAS-Smo* variant transgenic fly strains were generated by recombining the appropriate p*UAST-attB* transgenes into the 65B2 *attP* locus using the *PhiC31* system [25]. Flies carrying a chromosome 2 insertion of the *UAS-smo^3′UTR^-dsRNA* transgene were generated by standard P-element-mediated transgenesis. Other fly strains and their sources: *gprk2^del1^* and *gprk2^KO^* [16]; *ap-GAL4*, *nub-GAL4*, *dpp^10638^* (*dpp-LacZ*), *UAS-Dicer*, *tubP::GAL80^ts^* were from the Bloomington *Drosophila* Stock Center.

Anti-pSer^604^ and anti-pThr^610^/pThr^612^ phosphospecific antisera were generated by GenScript. Rabbits were immunized with phosphorylated peptides (KGRL{pS}ITLYNTHC or CSITLYN{pT}H{pT}DPVGL), and antibody was isolated from serum by modified peptide affinity column purification and unmodified peptide cross-adsorption. Anti-Smo antibody was raised in guinea pigs against the same His-tagged fragment of the Smo C-terminus (amino acids 560–1036) as previously used [26]. Other antibodies were: rabbit α-GFP (Torrey Pines Scientific); rabbit α-β-galactosidase (Santa Cruz Biotechnology); mouse α-Ptc (ApaI; developed by I. Guerrero) and mouse α-En (4D9; developed by C. Goodman) were obtained from the Developmental Studies Hybridoma Bank, created by the NICHD of the NIH and maintained at the Department of Biology, University of Iowa.

### Fly crosses and immunostainings

For expression of Smo^SD^ variants, flies were mated at 25°C and 0–48 h old offspring transferred to 29°C to inhibit GAL80^ts^ and activate *apGAL4*-dependent transgene expression. For experiments involving rescue of dsRNA-mediated Smo depletion, crosses included a *UAS-Dcr* transgene and were carried out at 27°C to maximize the *smo* dsRNA phenotype while minimizing the ectopic effects of transgenic Smo overexpression. For experiments in a *gprk2^KO^/gprk2^del1^* mutant background, flies were mated at 25°C and 0–48 h old offspring transferred to the restrictive temperature of 29°C [16]. For processing of adult wings, flies were collected in 50% ethanol/50% glycerol. After rinsing with water, wings were transferred into a drop of Faure's solution on glass slides and cover-slipped. For imaginal disc analyses, wandering third instar larval wing discs were dissected in phosphate-buffered saline (PBS) and kept on ice for a maximum of 20 min before fixation in PBS/0.2% Tween (PBT) containing 4% parafomaldehyde for 20 min. Discs were washed three times in PBT, followed by incubation for 30 min in PBT with 0.1% BSA (BBT). Primary antibodies were diluted in BBT, added to the discs and incubated over night at 4°C. After four washes with PBT, the discs were incubated with fluorescently-labeled secondary antibodies (Invitrogen and Jackson ImmunoResearch Laboratories) diluted in BBT, for 2 hours at room temperature. After four to five more washes with PBT, discs were mounted on slides in mounting medium (10% PBS, 90% glycerol, 0.2% n-propyl gallate), cover-slipped, and imaged using a Zeiss LSM700 confocal microscope.

### Genotypes

Fig. 3A - *UAS-Dcr/+;nub-GAL4/+*

Fig. 3B - *UAS-Dcr/UAS-GFP;nub-GAL4/UAS-smo^3′UTR^-dsRNA*

Fig. 3C - *UAS-Dcr/+;nub-GAL4/UAS-smo^3′UTR^-dsRNA;UAS-Smo^WT^/+*

Fig. 3D - *UAS-Dcr/+;nub-GAL4/UAS-smo^3′UTR^-dsRNA;UAS-Smo^c1-4A^/+*

Fig. 3F - *UAS-Dcr/+;nub-GAL4/UAS-smo^3′UTR^-dsRNA;UAS-Smo^core^/+*

Fig. 4A, B - *UAS-Dcr/UAS-GFP;ap-GAL4,dpp^10638^/UAS-smo^3′UTR^-dsRNA*

Fig. 4C, D, K - *UAS-Dcr/+;ap-GAL4,dpp^10638^/UAS-smo^3′UTR^-dsRNA;UAS-Smo^WT^-GFP/+*

Fig. 4E, F, L - *UAS-Dcr/+;ap-GAL4,dpp^10638^/UAS-smo^3′UTR^-dsRNA;UAS-Smo^c1-4A^-GFP/+*

Fig. 4G - *ap-GAL4/+;UAS-Smo^SD^-GFP/tubP::GAL80^ts^*

Fig. 4H - *ap-GAL4/+;UAS-Smo^SD.c1-4A^-GFP/tubP::GAL80^ts^*

Fig. 4I - *ap-GAL4/+;UAS-Smo^SD^-GFP,gprk2^del1^/gprk2^KO^*

Fig. 4J - *ap-GAL4/+;UAS-Smo^SD.c12D^-GFP,gprk2^del1^/gprk2^KO^*

Fig. 5D, E, I - *UAS-Dcr/+;ap-GAL4,dpp^10638^/UAS-smo^3′UTR^-dsRNA;UAS-Smo^core^-GFP/+*

Fig. 5F, G, J - *UAS-Dcr/+;ap-GAL4,dpp^10638^/UAS-smo^3′UTR^-dsRNA;UAS-Smo^core.c1-3A^-GFP/+*

Fig. 5H, K - *ap-GAL4/+;UAS-Smo^core^-GFP,gprk2^del1^/gprk2^KO^*

### Cell culture, dsRNA treatment, transfections, *ptc-luciferase* reporter assays and BRET experiments

Most experiments were performed using S2-R+ cells grown in *Drosophila* Schneider's medium (Lonza) supplemented with 10% fetal bovine serum (Gibco) and 50 U/ml penicillin and streptomycin (Gibco). Exceptionally, experiments for Figures 1B and C were performed using *Drosophila* S2 cells adapted to growth in serum-free medium (EX-CELL 420, Sigma), which show more pronounced Smo phosphoshifts due to a higher basal level of phosphorylation [19]. Cells were cultured at 25°C unless otherwise indicated. dsRNA was prepared by *in vitro* transcription using templates PCR-amplified from genomic DNA (nt 281756..281981 of genomic scaffold 2L for *smo* 3′-UTR; nt 372–870 of the *gprk2* coding sequence; nt 27259182..27259345 of genomic scaffold 3R for *gprk2* 5′-UTR; and nt 27282732..27283011 of genomic scaffold 3R for the 3′-UTR of *gprk2*). β-Gal dsRNA was used as a control. Forward and reverse primers included T7 (5′-TAATACGACTCACTATAGGGAGA-3′) and T3 (5′-AATTAACCCTCACTAAAGGGAGA-3′) promoter sequences, respectively. Top and bottom strand RNAs were generated using MEGAscript T7 and T3 *in vitro* transcription kits, mixed in equal amounts, and heated to 95°C followed by slow cooling to room temperature to anneal.

For biochemical analysis, <1×10^6^ cells were typically plated on day 1 in 24 well plates in 0.5 ml of complete Schneider's medium and each well was transfected with 100–250 ng of the indicated *pRmHa.puro* expression constructs using X-tremeGENE HP transfection reagent (Roche) according to manufacturer's instructions. On day 2, the cells of each well were split into 2 new wells of a 24 well plate and treated with 5 µg of the indicated dsRNA. On day 3 to 4, a second dose of dsRNA was applied and transgene expression was induced by addition of CuSO_4_ to a final concentration of 0.5 mM. Cells were harvested and processed on day 7.

For *ptc-luc* reporter assays, S2-R+ cells were transfected in 24 well plates on day 1 of the experiment as described above. 100 ng *pRmHa/Ci*, 75 ng *pGL.basic/ptcΔ136-luc* [22], 75 ng *pRL/CMV*, and 100 ng of each additional expression plasmid (Smo/Gprk2 variant; Hh^N^, as indicated) were typically used. Total DNA amounts in the transfection mix were normalized using empty *pRmHa.puro* vector. On day 2 the cells were split into 4 wells of a 96 well plate and each well was treated with 0.5–1 µg dsRNA. On day 3 or 4 transgene expression was induced by addition of CuSO_4_ and a second dose of dsRNA was administered. Cells were processed on day 7 and luciferase activity measured using the Dual Luciferase Reporter system (Promega) according to manufacturer's instructions. Assays were performed at least two times in quadruplicate, and the data was pooled. Statistical significance was assessed using two-tailed Student's *t*-tests.

For bioluminescence resonance energy transfer (BRET) experiments measuring Smo dimerization S2-R+ cells were transfected with 100 ng of the Smo^SD^-RLucII variant, 300 ng of the Smo-GFP10 variant and 100 ng of ^myc^Gprk2 (if applicable) per well of a 24-well plate. For BRET assays monitoring recruitment of Cos2 75 ng of Cos2-RLucII, 300 ng of the indicated Smo-GFP10 variant, and 75 ng ^myc^Fu plasmids were transfected. Cells were re-plated in 4 wells of a white-walled 96-well plate and subjected to dsRNA treatments and transgene induction as described above. BRET measurements were performed on day 7 as previously described [19]. Assays were performed at least two times in quadruplicate, and the data was pooled.

### Immunoprecipitations, SDS-PAGE, and immunoblotting

S2 cells expressing Smo-GFP variants were lysed in lysis buffer [50 mM Tris pH 7.5, 150 mM NaCl, 1% NP and containing 120 µg/ml AEBSF (Sigma), 1× protease inhibitors (Roche) and 1× phosphatase inhibitors (Roche)] for 15 min on ice. Insoluble material was removed by microcentrifugation for 15 min at 12,000× *g* and 4°C. Anti-GFP mAb agarose (MBL International) was added to soluble extracts and samples incubated on ice for 2 h. Beads were washed 2–3 times in 1 volume of lysis buffer and precipitated proteins extracted by addition of 1× SDS-PAGE sample buffer and heating at 75°C for 6 min. For most experiments, proteins were fractionated by SDS-PAGE on standard polyacrylamide gels. For Figure 1C, Phos-tag acrylamide (Wako Pure Chemicals Industries, Ltd.) was added to a final concentration of 7.5 mM to improve resolution of phosphoproteins [20]. Fractionated proteins were transferred to nitrocellulose membranes using a wet transfer apparatus and immunoblotted according to standard methods. Quantitation of signal intensity was performed using the Gels>Plot Lanes function of ImageJ 1.42q. Plots were normalized to equal total signal intensity (area under the curve), to correct for differences in loading.

### Immunoaffinity purification of Smo

S2-R+ cells were plated in 1 to 3 wells per condition of a 6-well plate and transfected with 2.5 µg/well of *pRmHa3.puro/Smo^SD^-GFP* as above. A day later, medium was replaced and 20 µg/well control (*β-gal*) or *gprk2* dsRNA was added to the cells. After three days of growth, the cells were harvested and replated in a 10-cm plate, along with 100 µg/plate of the appropriate dsRNA. Smo^SD^ expression was induced by addition of 0.5 mM CuSO_4_. 2–3 d later, cells were washed with ice-cold PBS and lysed in 3 ml RIPA buffer for 15 min on ice. Lysates were cleared by microcentrifugation for 15 min at 12,000× *g* and 4°C. Smo^SD^-GFP was immunoprecipitated using anti-GFP mAb agarose for 2 h at 4°C with rotation. Beads were washed 4 times with ice-cold RIPA buffer before addition of 1× SDS-PAGE sample buffer and heating at 75°C for 6 min. Samples were frozen at −80°C and typically 2 to 3 such preps were pooled for subsequent analysis. Pooled samples were fractionated by SDS-PAGE on 4–15% polyacrylamide gradient gels (BioRad), stained using Colloidal Blue (Life Technologies) according to manufacturer's protocol, and the band corresponding to Smo was excised from the gel.

### Protein digestion

Gel pieces were washed with water for 5 min and destained twice with the destaining buffer (50 mM ammonium bicarbonate, acetonitrile) for 15 min. An extra wash of 5 min was performed after destaining with a buffer of ammonium bicarbonate (50 mM). Gel pieces were then dehydrated with acetonitrile. Proteins were reduced by adding the reduction buffer (10 mM DTT, 100 mM ammonium bicarbonate) for 30 min at 40°C, and then alkylated by adding the alkylation buffer (55 mM iodoacetamide, 100 mM ammonium bicarbonate) for 20 min at 40°C. Gel pieces were dehydrated and washed at 40°C by adding ACN for 5 min before discarding all the reagents. Gel pieces were dried for 5 min at 40°C and then re-hydrated at 4°C for 40 min with enzyme solution. Tryptic digestion was performed with a 6 ng/µl solution of sequencing grade trypsin from Promega in 25 mM ammonium bicarbonate buffer, incubated at 58°C for 1 h and stopped with 15 µl of 1% formic acid/2% acetonitrile. Chymotryptic digestion was performed with a 40 ng/µl solution (Roche) in 100 mM Tris HCl- 25, mM CaCl2, pH 8 buffer, incubated at 25°C for 4 h and stopped with 15 µl of 1% formic acid/2% acetonitrile. Supernatant was transferred into a 96-well plate and peptide extraction was performed with two 30-min extraction steps at room temperature using the extraction buffer (1% formic acid/50% ACN). All peptide extracts were pooled into the 96-well plate and then completely dried in vacuum centrifuge. The plate was sealed and stored at −20°C until LC-MS/MS analysis. Protein digestion with Asp-N was performed in solution on tryptic digests. Samples were re-solubilized in a 50 mM ammonium bicarbonate buffer and 1 ng of Asp-N was added to each sample. Samples were incubated at 37°C for 3 h.

### Liquid chromatography/tandem mass spectrometry (LC-MS/MS) analysis

Prior to LC-MS/MS, peptide extracts were re-solubilized under agitation for 15 min in 11 µl of 0.2% formic acid and then centrifuged at 2000 rpm for 1 min. The LC column was a C18 reversed-phase column packed with a high-pressure packing cell. A 75 µm i.d. Self-Pack PicoFrit fused silica capillary (New Objective, Woburn, MA) of 15 cm length was packed with the C18 Jupiter 5 µm 300 Å reverse-phase material (Phenomenex, Torrence, CA). This column was installed on the Easy-nLC II system (Proxeon Biosystems, Odense, Denmark) and coupled to the LTQ Orbitrap Velos (ThermoFisher Scientific, Bremen, Germany) equipped with a Proxeon nanoelectrospray ion source. The buffers used for chromatography were 0.2% formic acid (buffer A) and 100% acetonitrile/0.2% formic acid (buffer B). During the first 12 min, 5 µl of sample were loaded on column with a flow of 600 nl/min and, subsequently, the gradient went from 2–80% buffer B in 60 min at a flow rate of 250 nL/min and then came back at 600 nL/min to 2% buffer B for 10min. LC-MS/MS data acquisition was accomplished using an eleven scan event cycle comprised of a full scan MS for scan event 1 acquired in the Orbitrap which enables high resolution/high mass accuracy analysis. The mass resolution for MS was set to 60,000 (at m/z 400) and used to trigger the ten additional MS/MS events acquired in parallel in the linear ion trap for the ten most intense ions. Mass over charge ratio range was from 360 to 2000 for MS scanning with a target value of 1,000,000 charges and from ∼1/3 of parent m/z ratio to 2000 for MS/MS scanning with a target value of 10,000 charges. The data–dependent scan events used a maximum ion fill time of 100 ms and 1 microscan. Target ions already selected for MS/MS were dynamically excluded for 25 s. Nanospray and S-lens voltages were set to 0.9–1.8 kV and 50 V, respectively. Capillary temperature was set to 225°C. MS/MS conditions were: normalized collision energy, 35 V; activation q, 0.25; activation time, 10 ms.

### Peptide identification and quantification

The peak list files were generated with extract_msn.exe (version January 10, 2011) using the following parameters: minimum mass set to 600 Da, maximum mass set to 6000 Da, no grouping of MS/MS spectra, precursor charge set to auto, and minimum number of fragment ions set to 10. MS/MS spectra were queried against the Smo^SD^ sequence using Mascot 2.3 (Matrix Science). The mass tolerances for precursor and fragment ions were set to 10 ppm and 0.6 Da, respectively. Search parameters allowed for up to two missed enzyme cleavages. Oxidation of methionine and phosphorylation of serine, threonine and tyrosine were allowed as variable modifications while carbamidomethyl was set as a fixed modification. Matches for phosphopeptides were validated manually. In a few cases (twice phosphorylated species of cluster 1 peptide W.AKRKDFEDKGRLSITLY.N in Chymotrypisin digest, once and twice phosphorylated species of the cluster 3 peptide R.MALTGAATGNSSSHGPR.K in trypsin+AspN digests), the phosphopeptides were not confirmed by MS2, but were detected in full scan with mass accuracies of less than 2 ppm, and eluted with very similar retention times to other phosphospecies of the same peptide. Peptides were quantitated by manual integration of precursor ion LC spectra using Qual Browser (Xcalibur from Thermo Scientific) [21], [41]. For each phosphopeptide identified, the relative level of phosphorylation in each sample was calculated as the ratio of the amount of phosphorylated: non-phosphorylated forms of the peptide.

### Smo sequence analysis

Multiple sequence alignment of full-length Smo proteins from nine bilaterian animal species was generated with Clustal-Omega. The species and accession numbers corresponding to the sequences used were: *Homo sapiens* (NP_005622.1), *Mus musculus* (NP_795970.3), *Danio rerio* (NP_571102.1), *Paracentrotus lividus* (AEX61000.1), *Platynereis dumerilii* (ADK38671.1), *Drosophila melanogaster* (NP_523443.1), *Apis mellifera* (XP_395373.3), *Tribolium castaneum* (NP_001127850.1), *Daphnia pulex* (EFX80809.1).

## Supporting Information

Figure S1 GPS1 and GPS2 are not the principal Gprk2 phosphorylation sites in Smo. (A) Western blot analysis of GFP immunoprecipitates from S2 cells expressing Smo^SD^-GFP or Smo^SD.GPSA12^-GFP, with or without Gprk2 depletion. The blot was probed with anti-GFP antibody to visualize tagged Smo protein. All bands are from the same exposure of a single blot with intervening lanes removed. Although Smo^SD.GPSA12^-GFP has putative Gprk2 phosphorylation sites mutated to nonphosphorylatable Ala, it still undergoes a similar phosphoshift as Smo^SD^-GFP in response to depletion of the kinase. (B and C) Confocal micrographs of wing discs with Smo variants - Smo^SD^-GFP (B) or Smo^SD.GPSA12^-GFP (C) - expressed in the dorsal compartment using *ap-GAL4*. Discs were immunostained to reveal En expression. Yellow dotted lines: A/P compartment boundaries based on domains of Ci expression (not shown). Both Smo^SD^-GFP and Smo^SD.GPSA12^-GFP drive comparable ectopic expression of En in dorsal anterior cells (arrowheads) - compare to wild-type ventral anterior cells. Genotypes: *ap-GAL4/+;UAS-Smo^SD^-GFP/tubP::GAL80^ts^* (*B*); *ap-GAL4/+;UAS-Smo^SD.GPSA12^-GFP/tubP::GAL80^ts^* (*C*).
(TIF)

Figure S2 Additional Gprk2 phosphorylation sites outside of clusters 1, 2, and 3 exist in Smo. Western blot analysis of GFP immunoprecipitates from S2 cells expressing Smo^SD^-GFP or Smo^SD.c1-3A^-GFP, with or without *gprk2* depletion. The blot was probed with anti-GFP antibody to visualize tagged Smo protein. All bands are from the same exposure of a single blot with intervening lanes removed. Smo^SD.c1-3A^-GFP migrates as a tighter band than Smo^SD^-GFP in control cells, suggesting that it is less phosphorylated. However, it still undergoes a phosphoshift in response to depletion of the kinase, indicating that additional Gprk2 phosphorylation sites exist.
(TIF)

Figure S3 Gprk2 promotes target gene expression downstream of Smo in S2-R+ cells in a catalytic activity-dependent manner. (A) Rescue of Smo^SD^-GFP-driven *ptc-luc* reporter activity in *gprk2*-depleted S2-R+ cells. Treatment of cells with *gprk2 5′*- and *3′-UTR* dsRNAs reduced *ptc-luc* reporter activity, and this was fully rescued by re-expressing wild-type Gprk2 but not kinase-dead Lys^338/339^→Met (kd1) or Asp^453^→Asn (kd2) mutants of Gprk2. (B) Western blot analysis of GFP immunoprecipitates (*top*) or total-cell lysates (*bottom*) of S2 cells with or without *gprk2* depletion, transfected with Smo^SD^-GFP along with empty vector (−) or various forms of Myc-tagged Gprk2. The blots were probed with anti-GFP (*top*) or anti-Myc (*bottom*) antibodies. Re-expression of wild-type (lane 4) but not kd1 (lane 6) or kd2 (lane 8) Gprk2 mutants rescued the Smo phosphoshift in Gprk2-depleted cells, confirming that the Gprk2 mutants are catalytically inactive. (C) Rescue of *ptc-luc* reporter activity in *gprk2*-depleted S2-R+ cells. Treatment of cells with *gprk2 5′-* and *3′-UTR* dsRNAs reduced Hh-dependent *ptc-luc* reporter activity, and this was rescued by re-expressing wild-type Gprk2. For this experiment, cells were cultured at the restrictive temperature for Gprk2 of 29°C.
(TIF)

Figure S4 Expression and cell surface accumulation of Gprk2 phosphosite cluster mutant forms of Smo. (A) Immunoblot analysis of Gprk2 phosphorylation cluster Ala mutant Smo^SD^-GFP variants from a *ptc-luc* reporter assay setup as in Figure 2A. Proteins were expressed at similar levels. (B) Immunoblot analysis of cell-surface wild-type Smo-GFP and Smo^c1-4A^-GFP in cells treated with or without Hh. Cell surface proteins were labeled by surface biotinylation. After lysis, biotinylated proteins were recovered by avidin-mediated affinity purification, and separated by SDS-PAGE. Smo was detected in the biotin-labeled surface protein fraction by immunoblotting with anti-GFP antibody. A background band served as a loading control to ensure that the starting samples had equivalent amounts of protein. Mutation of the Gprk2 phosphorylation sites did not impair the ability of Smo to reach the cell surface in response to Hh.
(TIF)

Figure S5 Multisite phosphorylation within Gprk2 phosphorylation clusters 1 and 2 is important for Smo activation. (A) *ptc-luc* reporter activity driven by Smo^SD^ variants with a subset of sites within clusters 1 or 2 mutated to Ala. Mutation of Ser^604^ and Thr^606^ (Smo^SD.c1AATT^) significantly reduced activity (**, Student *t*-test vs Smo^SD^, *p*<0.001), but not as much as mutation of all four residues (#, *t*-test versus Smo^SD.c1AATT^, *p*<0.001). Mutation of Ser^604^ and Thr^606^ individually (Smo^SD.c1ATTT^ and Smo^SD.c1SATT^) had much less effect than mutating both. Mutation of both Thr^610^ and Thr^612^ in cluster 1 (Smo^SD.c1STAA^) had no significant effect on Smo activity. The situation was similar for cluster 2, where mutating just three residues in either half of the cluster (Smo^SD.c2AAASST^ and Smo^SD.c1SSTAAA^) reduced activity (**, *t*-test vs Smo^SD^, p<0.001), but both had less effect than mutating all six (#, *t*-test versus Smo^SD.c2AAASST^ or Smo^SD.c2SSTAAA^, *p*<0.001). (B) Stimulation of Hh-dependent *ptc-luc* activity by Gprk2 phosphocluster mutants in Smo^WT^ backbone. Mutation all four cluster 1 or all six cluster 2 phosphorylation sites impairs Smo activity (**, *t*-test vs Smo^WT^, *p*<0.001). Cluster 3 or 4 mutations have no significant effect.
(TIF)

Figure S6 Expression analysis of C-terminally truncated Smo variants. Immunoblot analysis of the indicated C-terminally truncated Smo-GFP proteins from a *ptc-luc* reporter assay setup as in Figure 6D. Proteins were expressed at similar levels.
(TIF)

Figure S7 CKI does not phosphorylate the four mapped Gprk2 phosphorylation site clusters. (A) *ptc-luc* reporter activity in Hh-treated S2 cells is significantly reduced by treatment with dsRNA targeting CKI (*p*<.001), confirming that CKI was depleted. (B) Western blot analysis of GFP immunoprecipitates from S2 cells expressing Smo^SD^-GFP and treated with control, *gprk2*, or *ck1* dsRNA. The blot was probed with the Smo anti-pT^610^/pT^612^ phosphospecific antiserum (top) or with anti-Smo (bottom). Whereas Gprk2 depletion reduced bulk Smo phosphorylation and T^610^/T^612^ phosphorylation, CKI depletion had no discernible effect.
(TIF)

Table S1 Quantification of phosphorylated and matching non-phosphorylated Smo^SD^ peptides from LC-MS/MS analysis. Values represent area-under-the-curve measurements of precursor ion LC spectra (in arbitrary units).
(DOC)
