# Supplementary figures and images for: A Broadly Conserved G-Protein-Coupled Receptor Kinase Phosphorylation Mechanism Controls Drosophila Smoothened Activity
Source: PLoS Genet. 2014 Jul 10;10(7):e1004399. doi: 10.1371/journal.pgen.1004399 (PMC4091690; doi:10.1371/journal.pgen.1004399)

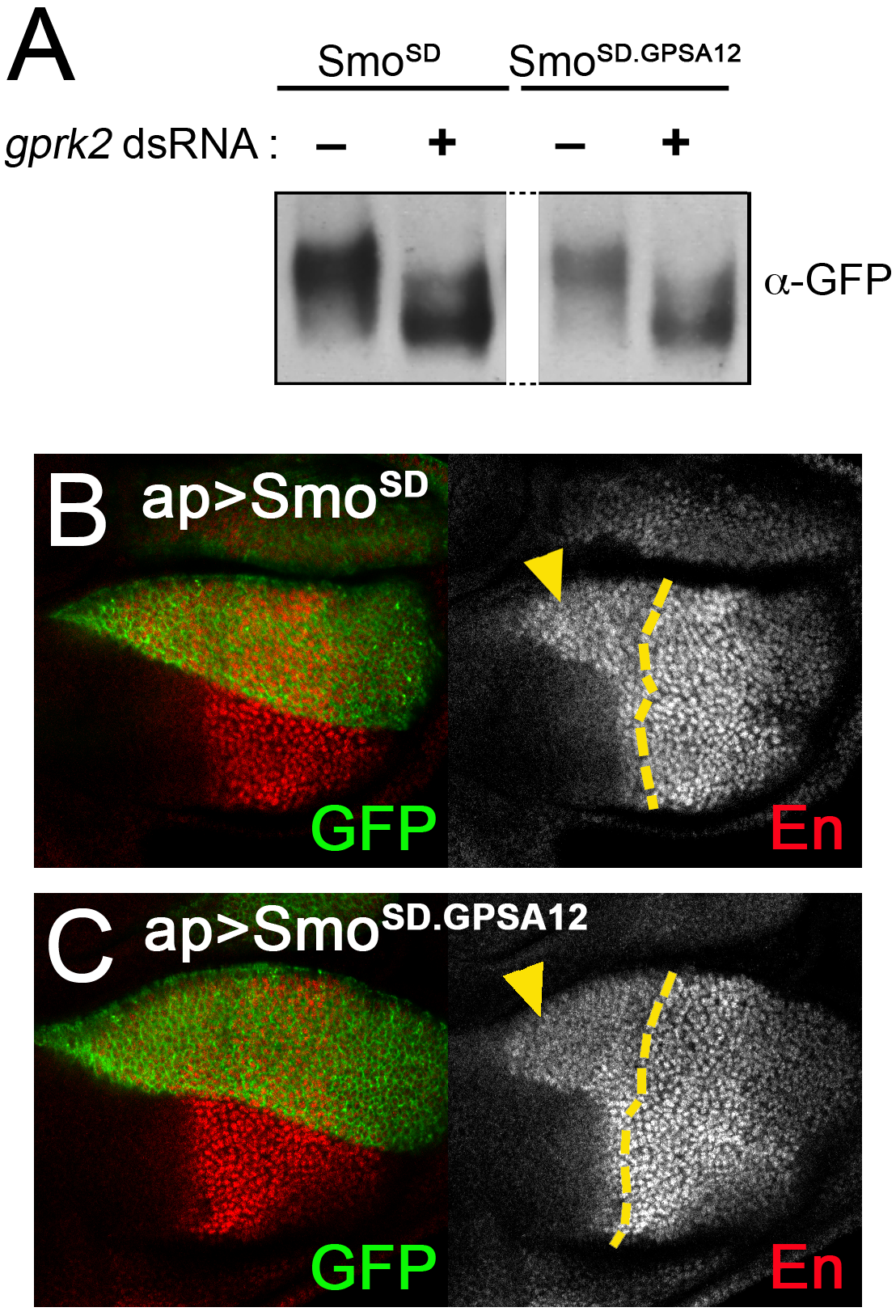

Supplement: Figure S1 — GPS1 and GPS2 are not the principal Gprk2 phosphorylation sites in Smo. (A) Western blot analysis of GFP immunoprecipitates from S2 cells expressing SmoSD-GFP or SmoSD.GPSA12-GFP, with or without Gprk2 depletion. The blot was probed with anti-GFP antibody to visualize tagged Smo protein. All bands are from the same exposure of a single blot with intervening lanes removed. Although SmoSD.GPSA12-GFP has putative Gprk2 phosphorylation sites mutated to nonphosphorylatable Ala, it still undergoes a similar phosphoshift as SmoSD-GFP in response to depletion of the kinase. (B and C) Confocal micrographs of wing discs with Smo variants - SmoSD-GFP (B) or SmoSD.GPSA12-GFP (C) - expressed in the dorsal compartment using ap-GAL4. Discs were immunostained to reveal En expression. Yellow dotted lines: A/P compartment boundaries based on domains of Ci expression (not shown). Both SmoSD-GFP and SmoSD.GPSA12-GFP drive comparable ectopic expression of En in dorsal anterior cells (arrowheads) - compare to wild-type ventral anterior cells. Genotypes: ap-GAL4/+;UAS-SmoSD-GFP/tubP::GAL80ts (B); ap-GAL4/+;UAS-SmoSD.GPSA12-GFP/tubP::GAL80ts (C). (TIF) [file pgen.1004399.s001.tif]

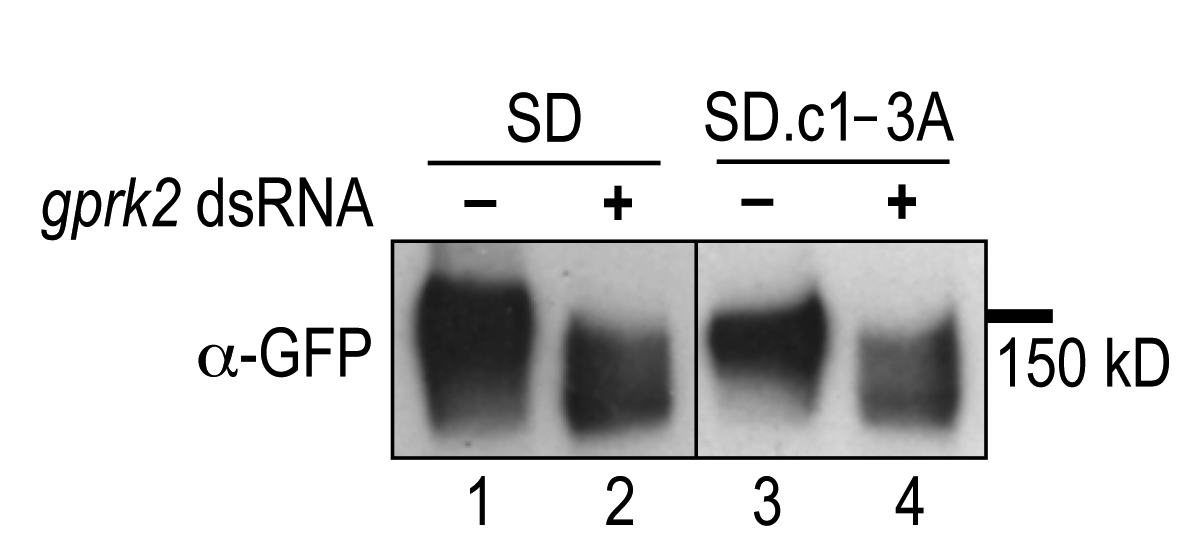

Supplement: Figure S2 — Additional Gprk2 phosphorylation sites outside of clusters 1, 2, and 3 exist in Smo. Western blot analysis of GFP immunoprecipitates from S2 cells expressing SmoSD-GFP or SmoSD.c1-3A-GFP, with or without gprk2 depletion. The blot was probed with anti-GFP antibody to visualize tagged Smo protein. All bands are from the same exposure of a single blot with intervening lanes removed. SmoSD.c1-3A-GFP migrates as a tighter band than SmoSD-GFP in control cells, suggesting that it is less phosphorylated. However, it still undergoes a phosphoshift in response to depletion of the kinase, indicating that additional Gprk2 phosphorylation sites exist. (TIF) [file pgen.1004399.s002.tif]

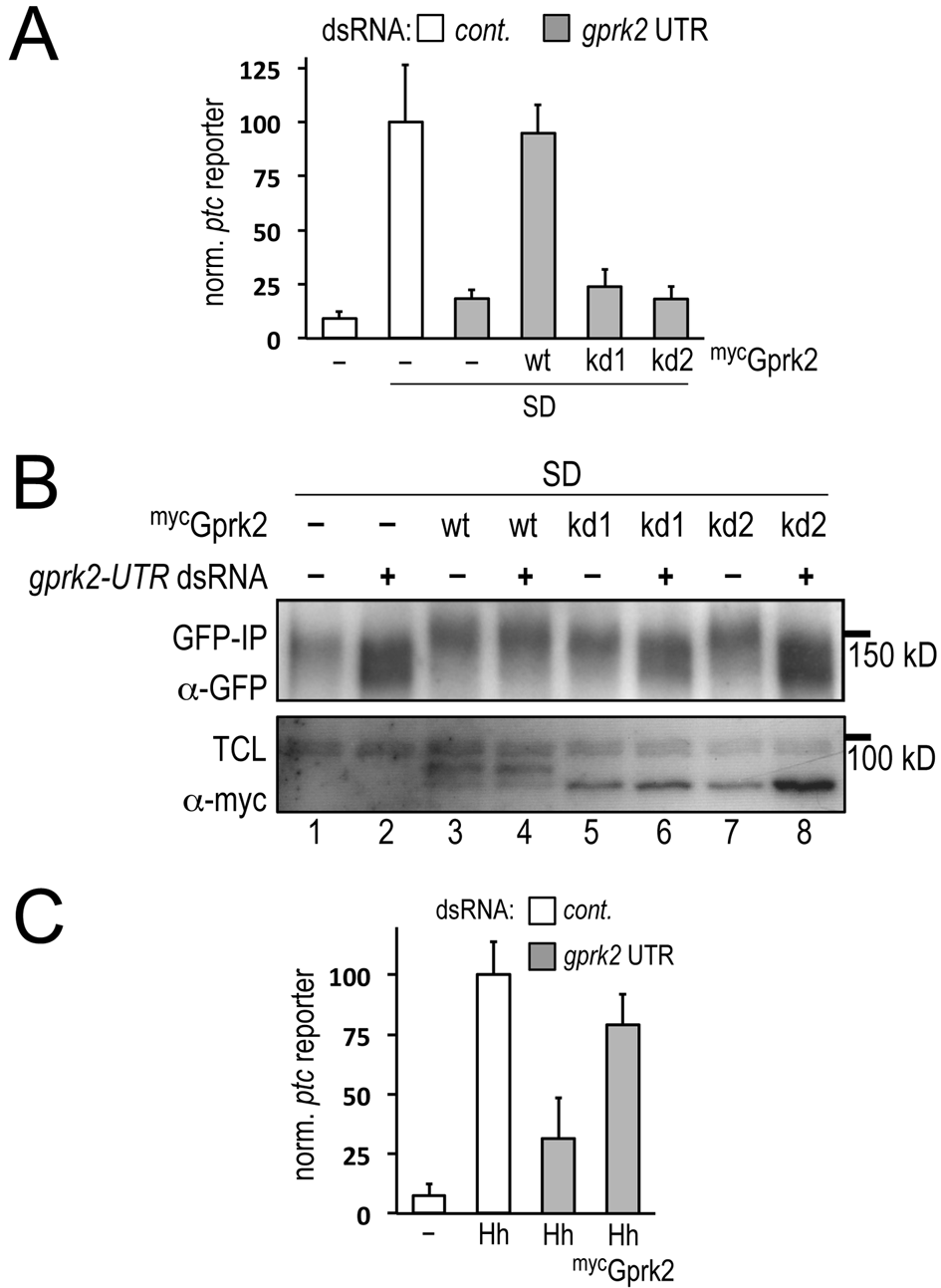

Supplement: Figure S3 — Gprk2 promotes target gene expression downstream of Smo in S2-R+ cells in a catalytic activity-dependent manner. (A) Rescue of SmoSD-GFP-driven ptc-luc reporter activity in gprk2-depleted S2-R+ cells. Treatment of cells with gprk2 5′- and 3′-UTR dsRNAs reduced ptc-luc reporter activity, and this was fully rescued by re-expressing wild-type Gprk2 but not kinase-dead Lys338/339→Met (kd1) or Asp453→Asn (kd2) mutants of Gprk2. (B) Western blot analysis of GFP immunoprecipitates (top) or total-cell lysates (bottom) of S2 cells with or without gprk2 depletion, transfected with SmoSD-GFP along with empty vector (−) or various forms of Myc-tagged Gprk2. The blots were probed with anti-GFP (top) or anti-Myc (bottom) antibodies. Re-expression of wild-type (lane 4) but not kd1 (lane 6) or kd2 (lane 8) Gprk2 mutants rescued the Smo phosphoshift in Gprk2-depleted cells, confirming that the Gprk2 mutants are catalytically inactive. (C) Rescue of ptc-luc reporter activity in gprk2-depleted S2-R+ cells. Treatment of cells with gprk2 5′- and 3′-UTR dsRNAs reduced Hh-dependent ptc-luc reporter activity, and this was rescued by re-expressing wild-type Gprk2. For this experiment, cells were cultured at the restrictive temperature for Gprk2 of 29°C. (TIF) [file pgen.1004399.s003.tif]

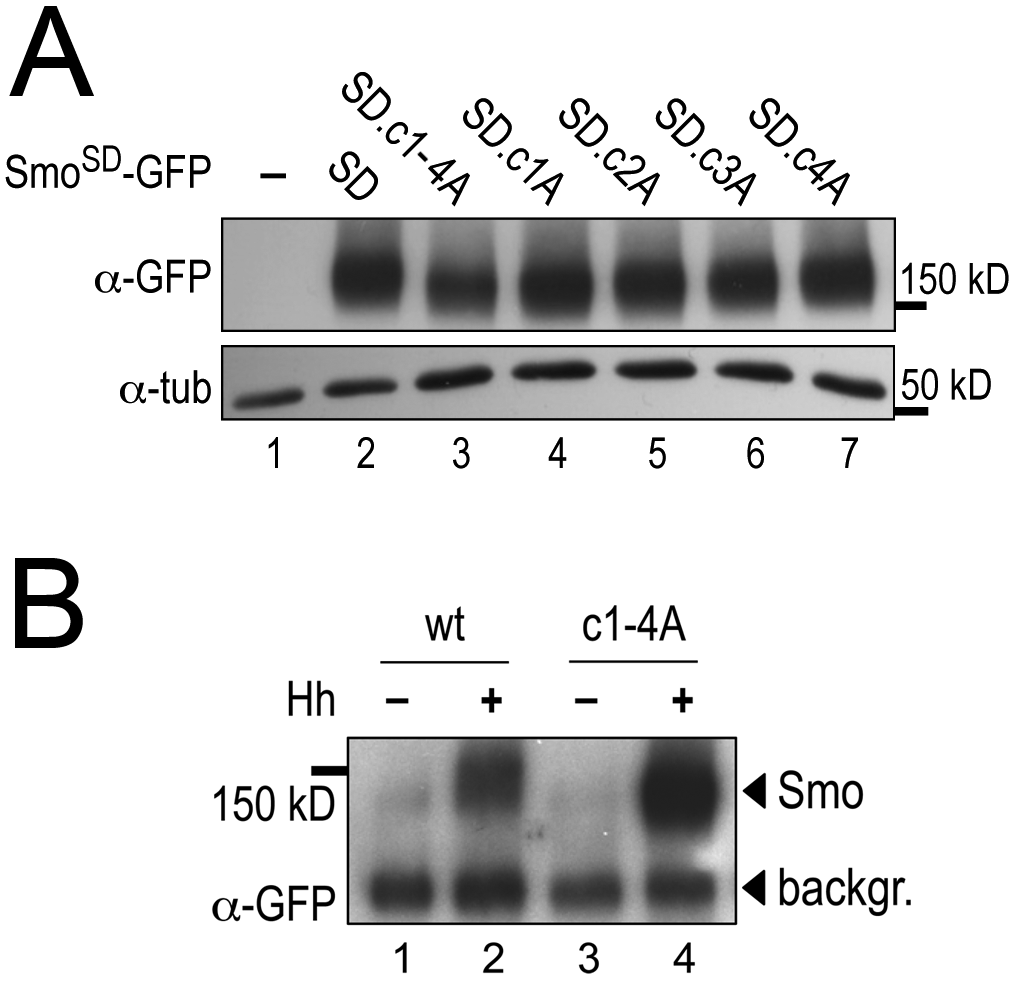

Supplement: Figure S4 — Expression and cell surface accumulation of Gprk2 phosphosite cluster mutant forms of Smo. (A) Immunoblot analysis of Gprk2 phosphorylation cluster Ala mutant SmoSD-GFP variants from a ptc-luc reporter assay setup as in Figure 2A. Proteins were expressed at similar levels. (B) Immunoblot analysis of cell-surface wild-type Smo-GFP and Smoc1-4A-GFP in cells treated with or without Hh. Cell surface proteins were labeled by surface biotinylation. After lysis, biotinylated proteins were recovered by avidin-mediated affinity purification, and separated by SDS-PAGE. Smo was detected in the biotin-labeled surface protein fraction by immunoblotting with anti-GFP antibody. A background band served as a loading control to ensure that the starting samples had equivalent amounts of protein. Mutation of the Gprk2 phosphorylation sites did not impair the ability of Smo to reach the cell surface in response to Hh. (TIF) [file pgen.1004399.s004.tif]

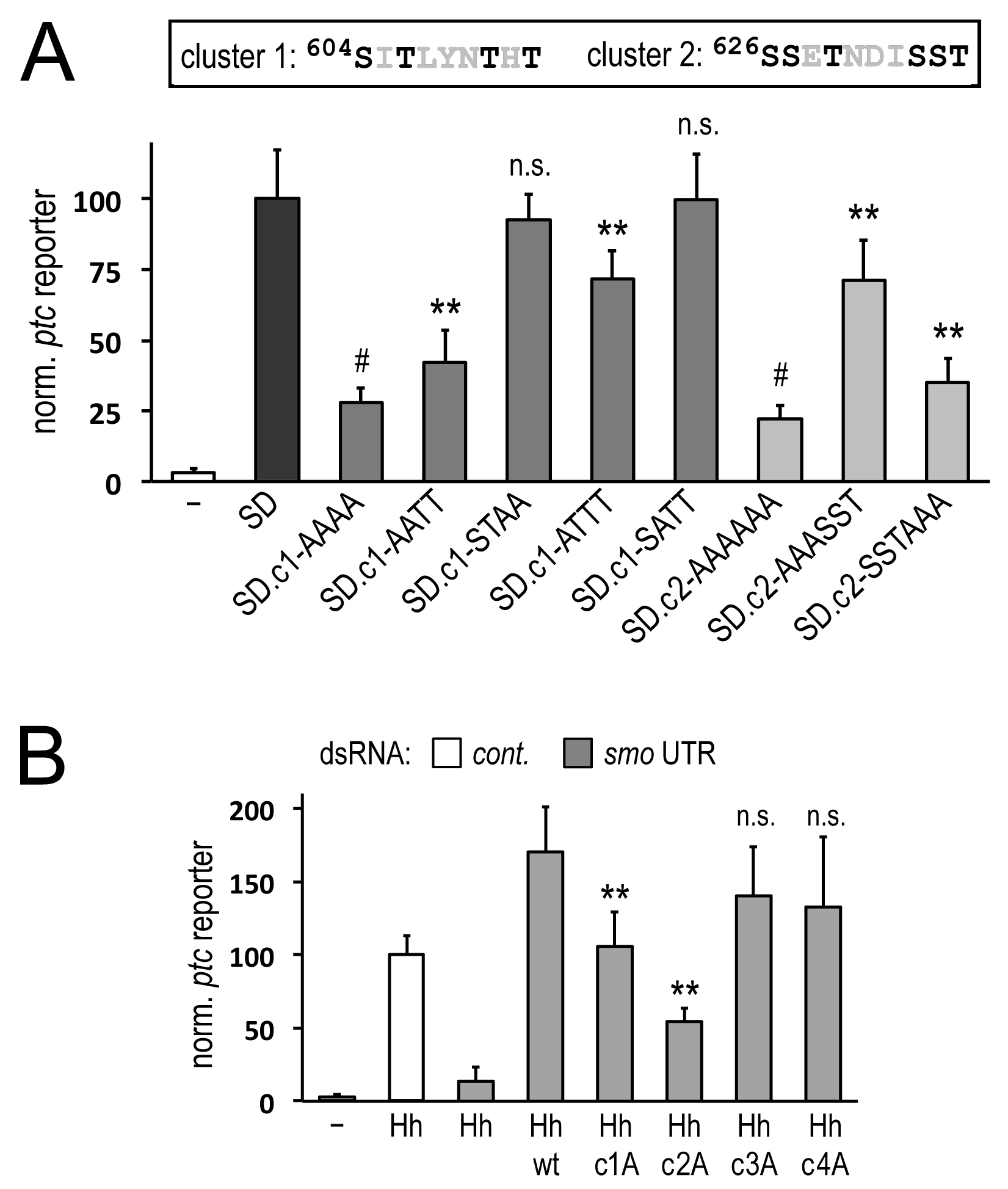

Supplement: Figure S5 — Multisite phosphorylation within Gprk2 phosphorylation clusters 1 and 2 is important for Smo activation. (A) ptc-luc reporter activity driven by SmoSD variants with a subset of sites within clusters 1 or 2 mutated to Ala. Mutation of Ser604 and Thr606 (SmoSD.c1AATT) significantly reduced activity (**, Student t-test vs SmoSD, p<0.001), but not as much as mutation of all four residues (#, t-test versus SmoSD.c1AATT, p<0.001). Mutation of Ser604 and Thr606 individually (SmoSD.c1ATTT and SmoSD.c1SATT) had much less effect than mutating both. Mutation of both Thr610 and Thr612 in cluster 1 (SmoSD.c1STAA) had no significant effect on Smo activity. The situation was similar for cluster 2, where mutating just three residues in either half of the cluster (SmoSD.c2AAASST and SmoSD.c1SSTAAA) reduced activity (**, t-test vs SmoSD, p<0.001), but both had less effect than mutating all six (#, t-test versus SmoSD.c2AAASST or SmoSD.c2SSTAAA, p<0.001). (B) Stimulation of Hh-dependent ptc-luc activity by Gprk2 phosphocluster mutants in SmoWT backbone. Mutation all four cluster 1 or all six cluster 2 phosphorylation sites impairs Smo activity (**, t-test vs SmoWT, p<0.001). Cluster 3 or 4 mutations have no significant effect. (TIF) [file pgen.1004399.s005.tif]

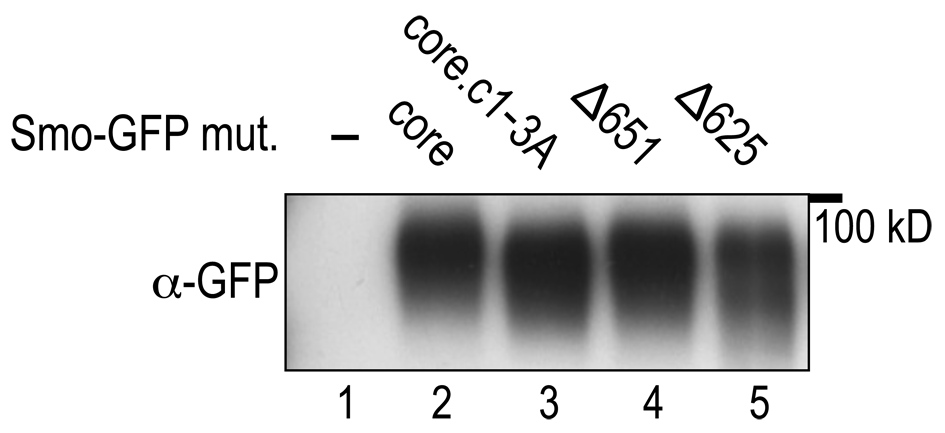

Supplement: Figure S6 — Expression analysis of C-terminally truncated Smo variants. Immunoblot analysis of the indicated C-terminally truncated Smo-GFP proteins from a ptc-luc reporter assay setup as in Figure 6D. Proteins were expressed at similar levels. (TIF) [file pgen.1004399.s006.tif]

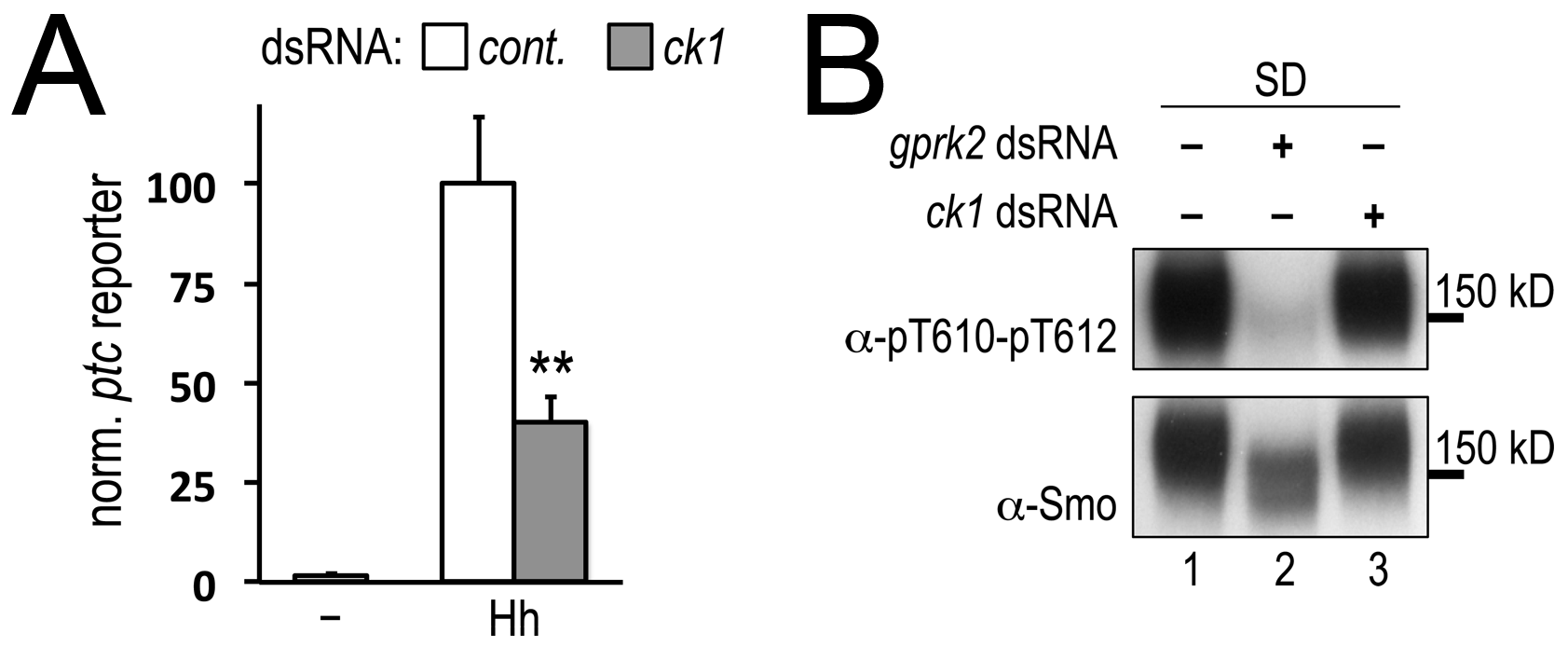

Supplement: Figure S7 — CKI does not phosphorylate the four mapped Gprk2 phosphorylation site clusters. (A) ptc-luc reporter activity in Hh-treated S2 cells is significantly reduced by treatment with dsRNA targeting CKI (p<.001), confirming that CKI was depleted. (B) Western blot analysis of GFP immunoprecipitates from S2 cells expressing SmoSD-GFP and treated with control, gprk2, or ck1 dsRNA. The blot was probed with the Smo anti-pT610/pT612 phosphospecific antiserum (top) or with anti-Smo (bottom). Whereas Gprk2 depletion reduced bulk Smo phosphorylation and T610/T612 phosphorylation, CKI depletion had no discernible effect. (TIF) [file pgen.1004399.s007.tif]
