# Supplementary material for: A Broadly Conserved G-Protein-Coupled Receptor Kinase Phosphorylation Mechanism Controls Drosophila Smoothened Activity
Source: PLoS Genet. 2014 Jul 10;10(7):e1004399. doi: 10.1371/journal.pgen.1004399 (PMC4091690; doi:10.1371/journal.pgen.1004399)
Supplement: Table S1 — Quantification of phosphorylated and matching non-phosphorylated SmoSD peptides from LC-MS/MS analysis. Values represent area-under-the-curve measurements of precursor ion LC spectra (in arbitrary units). (DOC) [file pgen.1004399.s008.doc]

TABLE S1. Quantification of Smo^SD^ unmodified and phosphopeptides by LC-MS/MS

|  | **Phosphosite** | **CONTROL dsRNA** | | **Gprk2 dsRNA** | | **RATIO CON:Gprk2** |
| --- | --- | --- | --- | --- | --- | --- |
|  |  | **Peptide quantity** | **phos/un-mod peptide** | **Peptide quantity** | **phos/un-mod peptide** |  |
| **CHYMOTRYPSIN** | | | | | | |
| cluster 1 | none | 5.96E+05 |  | 3.07E+06 |  |  |
|  | **p**S604 | 1.70E+06 | 2.852 | 8.76E+05 | 0.285 | **10.0** |
|  | **p**S604, **p**T606 | 2.31E+05 | 0.388 | 1.99E+05 | 0.065 | **6.0** |
| cluster 1 | none | 7.91E+07 |  | 1.09E+08 |  |  |
|  | **p**T610 | 3.52E+06 | 0.045 | 5.50E+04 | 0.001 | **88.2** |
|  | **p**T612 | 8.54E+06 | 0.108 | 4.92E+05 | 0.005 | **23.9** |
|  | **p**T610, **p**T612 | 1.89E+07 | 0.239 | 5.02E+05 | 0.005 | **51.9** |
| GPS1 | none | 1.62E+07 |  | 1.99E+07 |  |  |
|  | **p**S740  **p**T741 | 7.96E+06 | 0.492 | 4.92E+06 | 0.248 | **2.0** |
|  | **p**S740, **p**T741 | 3.46E+06 | 0.214 | 8.98E+05 | 0.045 | **4.7** |
|  |  |  |  |  |  |  |
| **TRYPSIN** | | | | | | |
| cluster 3 | none | 1.14E+09 |  | 1.32E+09 |  |  |
|  | **p**S658  **p**S659  **p**S660 | 1.36E+08 | 0.120 | 1.35E+07 | 0.010 | **11.7** |
|  | **p**S658, **p**S659  **p**S658, **p**S660  **p**S659, **p**S660 | 1.13E+06 | 0.001 | 1.01E+05 | 0.000 | **13.0** |
| cluster 4 | none | 7.31E+08 |  | 8.25E+08 |  |  |
|  | **p**S675 | 1.73E+08 | 0.236 | 4.14E+07 | 0.050 | **4.7** |
|  | **p**S675, **p**S680 | 6.08E+07 | 0.083 | 2.55E+06 | 0.003 | **26.9** |
|  | **p**S675, **p**S680, **p**S683 | 3.31E+07 | 0.045 | 1.33E+06 | 0.002 | **28.0** |
| cluster 4 | none | 1.87E+08 |  | 2.36E+08 |  |  |
|  | **p**S680 | 7.07E+06 | 0.038 | 7.61E+05 | 0.003 | **11.7** |
|  | **p**S680, **p**S683 | 2.07E+08 | 1.107 | 2.13E+07 | 0.090 | **12.2** |
| GPS1 | none | 2.53E+08 |  | 2.52E+08 |  |  |
|  | **p**S740 | 1.86E+07 | 0.074 | 9.80E+06 | 0.039 | **1.9** |
|  | **p**T741 | 1.40E+08 | 0.555 | 1.11E+08 | 0.441 | **1.3** |
|  | **p**S740, **p**T741 | 6.72E+07 | 0.266 | 2.06E+07 | 0.082 | **3.2** |
|  |  |  |  |  |  |  |
| **TRYPSIN + ASPN A** | | | | | | |
| cluster 3 | None | 2.28E+06 |  | 1.39E+06 |  |  |
|  | **p**S658  **p**S659  **p**S660 | 3.03E+05 | 0.133 | 2.70E+04 | 0.019 | **6.8** |
|  | **p**S658, **p**S659  **p**S658, **p**S660  **p**S659, **p**S660 | 1.81E+05 | 0.079 | 1.00E+04 | 0.007 | **11.0** |
|  | **p**S658, **p**S659, **p**S660 | 2.00E+05 | 0.088 | 1.00E+04 | 0.007 | **12.2** |
| cluster 4 | **p**S675, **p**S680 | 1.74E+06 |  | ND |  | ***** |
| cluster 4 | none | 1.50E+08 |  | 7.35E+07 |  |  |
|  | **p**S680 | 2.28E+07 | 0.152 | 1.50E+06 | 0.020 | **7.4** |
|  | **p**S680, **p**S683 | 5.30E+06 | 0.035 | 7.60E+04 | 0.001 | **34.2** |
| GPS1 | none | 4.61E+06 |  | 1.26E+06 |  |  |
|  | **p**S740 | 8.71E+06 | 0.189 | 2.59E+05 | 0.206 | **0.9** |
|  | **p**T741 | 1.27E+06 | 0.275 | 1.47E+05 | 0.117 | **2.4** |
|  | **p**S740, **p**T741 | 2.67E+06 | 0.579 | 1.47E+05 | 0.117 | **5.0** |
|  |  |  |  |  |  |  |
| **TRYPSIN + ASPN B** | | | | | | |
| cluster 3 | none | 1.09E+06 |  | 2.10E+06 |  |  |
|  | **p**S658  **p**S659  **p**S660 | 2.41E+05 | 0.221 | 5.20E+04 | 0.025 | **8.9** |
|  | **p**S658, **p**S659  **p**S658, **p**S660  **p**S659, **p**S660 | 8.51E+04 | 0.078 | 1.00E+04 | 0.005 | **16.4** |
|  | **p**S658, **p**S659, **p**S660 | 4.58E+04 | 0.042 | 1.00E+04 | 0.005 | **8.8** |
| cluster 4 | **p**S675, **p**S680 | 4.46E+05 |  | ND |  | ***** |
| cluster 4 | none | 8.23E+07 |  | 1.10E+08 |  |  |
|  | **p**S680 | 3.58E+06 | 0.043 | 1.51E+06 | 0.014 | **3.2** |
|  | **p**S680, **p**S683 | 4.10E+06 | 0.050 | 7.77E+05 | 0.007 | **7.1** |
| GPS1 | none | 1.45E+06 |  | 4.74E+05 |  |  |
|  | **p**S740 | 2.33E+05 | 0.161 | 3.64E+05 | 0.131 | **1.2** |
|  | **p**T741 | 2.65E+06 | 1.828 | 3.24E+06 | 1.165 | **1.6** |
|  | **p**S740, **p**T741 | 1.50E+06 | 1.034 | 1.99E+05 | 0.420 | **2.4** |
